# Crannenols A–D, Sesquiterpenoids from the Irish Deep-Sea Soft Coral *Acanella arbuscula*

**DOI:** 10.1021/acs.jnatprod.2c00602

**Published:** 2022-09-19

**Authors:** Joshua T. Welsch, Ryan M. Young, A. Louise Allcock, Mark P. Johnson, Bill J. Baker

**Affiliations:** † Department of Chemistry, University of South Florida, 4202 E. Fowler Avenue, CHE205, Tampa, Florida 33620, United States; ‡ School of Natural Sciences and Ryan Institute, University of Galway, University Road, Galway, H91 TK33, Ireland

## Abstract

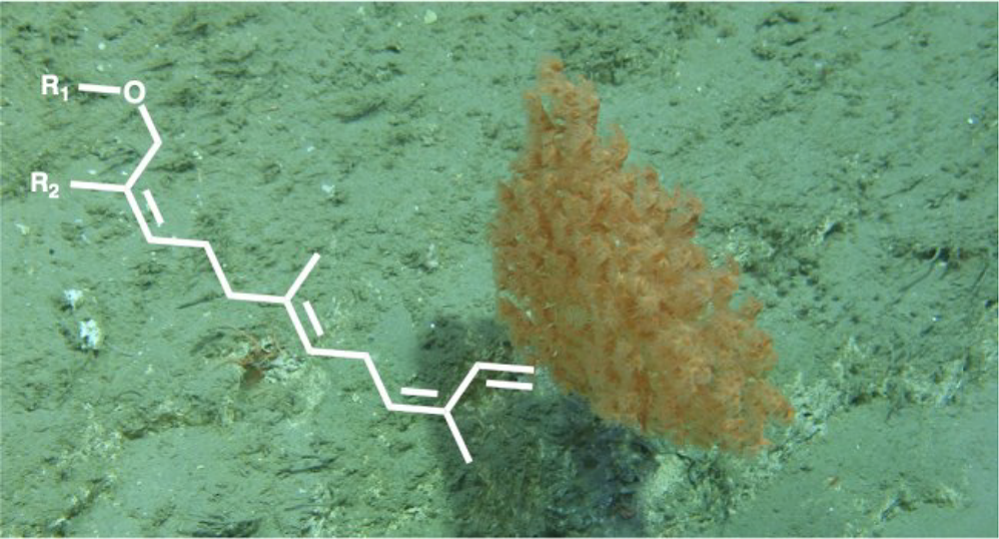

Four undescribed sesquiterpenoids, crannenols A–D (**1**–**4**), have been isolated from CHCl_2_ and MeOH extracts of the deep-sea bamboo coral *Acanella arbuscula*. The corals were collected from a submarine canyon on the edge of Ireland’s Porcupine Bank via a remotely operated vehicle. The structure elucidation of these (*Z*,*E*)-α-farnesene derivatives was achieved using a combination of 1D and 2D NMR, electron impact (**1**, **2**), and electrospray ionization (**3**, **4**) mass spectrometry.

Natural products (NPs) remain a vital point of inspiration for the development of modern medicines with roughly 50% of newly approved drugs in the last 40 years deriving their roots from secondary metabolites extracted from nature.^1^ Among more than 200 000 known NPs, only ∼30 000 derive from the marine environment and are primarily from organisms living in shallow, temperate, and tropical waters, despite roughly 93% of the oceans existing at depths greater than 1000 m, highlighting the need for investigation of biota from the deep.^2,3^ Technological advances including remotely operated vehicles (ROVs) and manned submersibles have afforded researchers new opportunities for targeted collections of such organisms at previously inaccessible depths.

Deep-sea octocorals of the order Alcyonacea are known producers of diverse terpenoids that often possess notable bioactivity; examples include the cytotoxic diterpene alcyonolide and the illuldalene sesquiterpenoids alcyopterosins.^4−6^ The current study was carried out to investigate the chemical diversity of the Irish deep-sea bamboo coral *Acanella arbuscula*, a species from which no prior chemical investigation has been reported.

## Results and Discussion

Dichloromethane and methanol extracts were subjected to a panel of bioassays where preliminary data indicated inhibitory activity against the bacteria *Clostridium difficile* and *Mycobacterium tuberculosis*, as well as respiratory syncytial virus. Repeat rounds of fractionation altering between normal and reversed phase chromatography yielded four new sesquiterpenoid (Z*,E*)-α-farnesene derivatives, crannenols A–D (**1**–**4**). Deriving their name from the Irish word “crann” meaning tree due to the branching resemblance of *A. arbuscula* to that of a small tree, the isolation and subsequent structure determination of compounds **1**–**4** are described herein.
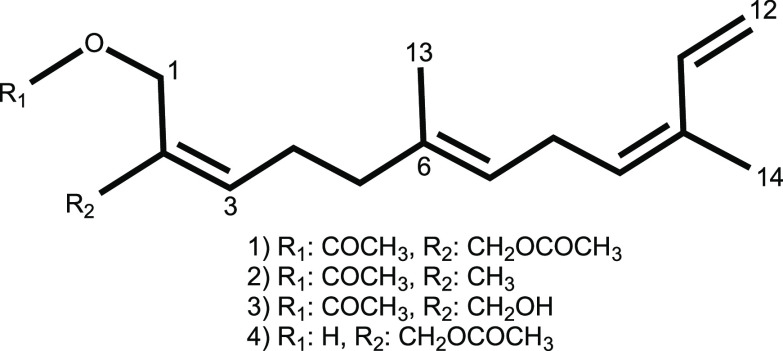


**Figure 1:**
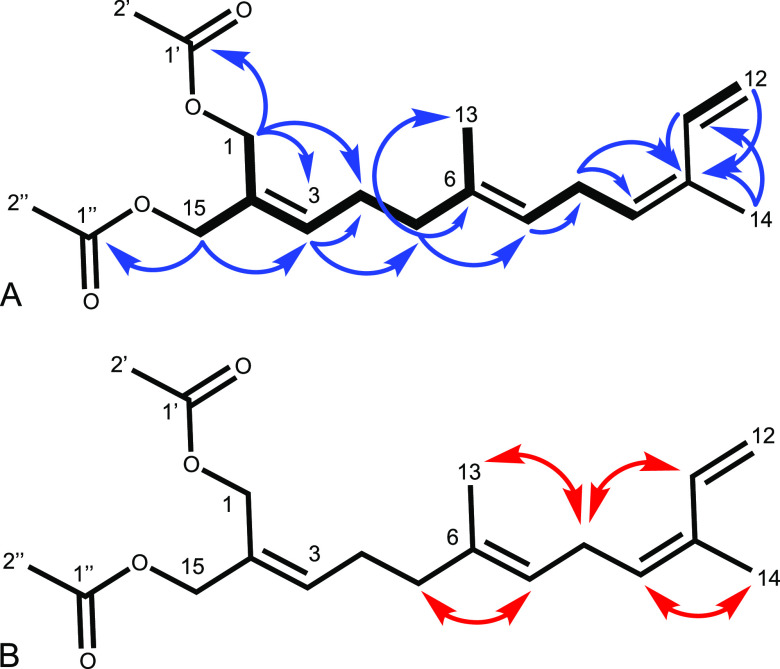
Key HMBC (blue →) and COSY (thick **―**) correlations (A) and key NOESY (red ↔) correlations (B) establishing the configuration of crannenol A (**1**).

Crannenol A (**1**) was isolated as a clear oil. A molecular formula of C_19_H_28_O_4_ was established by HREIMS corroborated by signals in the ^1^H and ^13^C NMR spectra (Table 1). Key ^1^H NMR signals (Figure 1a) included two acetoxy-bearing methylene singlets H_2_-1 and H_2_-15 (δ_H_ 4.65 and 4.56, respectively), which demonstrated long-range (allylic and W) coupling in the COSY spectrum to each other as well as through C-2 (δ_C_ 129.1) to H-3 (δ_H_ 5.75) (Figure 1a). H_2_-1 and H_2_-15 both showed HMBC correlations to their respective acetate carbonyls C-1′ (δ_C_ 170.9) and C-1″ (δ_C_ 170.7), as well as to C-3 (δ_C_ 136.1), with H_2_-1 extending a four-bond correlation to C-4 (δ_C_ 26.1). COSY correlations of H-3 linked to both quartet and triplet methylenes in H_2_-4 (δ_H_ 2.26) and H_2_-5 (δ_H_ 2.06), respectively, which were confirmed by HMBC correlations of H-3 to C-4 and C-5 (δ_C_ 38.9). H_2_-5 displayed a further COSY correlation to a singlet methyl, H_3_-13 (δ_H_ 1.64), as well as the corresponding HMBC correlation to C-13 (δ_C_16.0), and additional correlations to the vinyl olefin C-6 (δ_C_ 134.4) and methine C-7 (δ_C_ 123.4), establishing a trisubstituted alkene. H-7 (δ_H_ 5.13) was shown to correlate in the COSY spectrum to the triplet methylene H_2_-8 (δ_H_ 2.87) and triplet methine H-9 (δ_H_ 5.34), corroborated with an HMBC correlation from H-7 to C-8 (δ_C_ 26.3). H_2_-8 showed a correlation in the COSY spectrum only to H-9, yet displayed HMBC correlations to both C-9 (δ_C_129.3) and C-10 (δ_C_ 132.1), indicative of C-10 as a quaternary olefin. C-10 correlated in the HMBC spectrum with the singlet methyl H_3_-14 (δ_H_ 1.82) as well as the methine H-11 (δ_H_ 6.80), displaying a doublet-of-doublets splitting pattern, and two doublets accounting for the terminal olefinic protons of H_2_-12 (δ_H_ 5.11 and 5.21). The proximity of H-11 and H-12 was further confirmed by a COSY correlation between the two. The double-bond configurations of crannenol A were determined using 2D NOESY NMR correlations (Figure 1b), which demonstrated proximity of protons H_2_-5/H-7 and H_2_-8/H_3_-13, suggesting the *E* configuration for the C-6/C-7 olefin. The C-9/C-10 olefin was assigned the *Z* configuration based on the observations of correlations between H_2_-8/H-11 and H-9/H_3_-14.

**Table 1:** NMR Data for Crannenol A (**1**)^a^

pos	δ_C_,^b^ type	δ_H_^c^ (*J* in Hz)	HMBC^c^	COSY^c^	NOESY^c^
1	59.8, CH_2_	4.65, s	3, 4, 1′	3, 15	
2	129.1, C				
3	136.1, CH	5.75, t (7.4)	4, 5	4, 5	
4	26.1, CH_2_	2.26, q (7.6)			13
5	38.9, CH_2_	2.06, m	6, 7, 13	13	7
6	134.4, C				
7	123.4, CH	5.13, t	8	8, 9	
8	26.3, CH_2_	2.87, t (7.3)	9, 10	9	13, 11
9	129.3, CH	5.34, t (7.5)			14
10	132.1, C				
11	133.5, CH	6.80, dd (17.3, 10.8)	10	12	
12	113.6, CH_2_	5.11, d (10.7)	10		
		5.21, d (17.2)	10		
13	16.0, CH_3_	1.64, s			
14	19.7, CH_3_	1.82, s	10, 11		
15	66.7, CH_2_	4.56, s	3, 1″		
1′	170.9, C				
1″	170.7, C				
2′	20.9, CH_3_	2.07, s			
2″	21.0, CH_3_	2.07, s			

a CDCl_3_, ppm, multiplicity determined by HSQC.

b 150 MHz.

c 600 MHz.

Crannenol B (**2**) was isolated as a clear oil with spectroscopic data similar to that of crannenol A (**1**). A molecular formula of C_17_H_26_O_2_ for compound **2** was established from HREIMS corroborated by ^1^H and ^13^C NMR spectra (Table 2). Crannenol B departed from the motif of **1** by displaying only a single acetate methyl group in the ^1^H NMR spectrum. A new singlet methyl present in **2**, H_3_-15 (δ_H_ 1.75), was confirmed through COSY correlations of H_3_-15 to both the triplet methine H-3 (δ_H_ 5.38) and singlet methylene H_2_-1 (δ_H_ 4.58), as well as HMBC correlations of H_3_-15 to olefinic carbons C-2 (δ_C_ 129.8) and C-3 (δ_C_ 130.5), and the acetoxy-bearing methylene C-1 (δ_C_ 63.2). The remainder of the carbon skeleton of **2** was determined to mirror that of **1** on the basis of ^1^H and ^13^C NMR data. The configuration of the C-2/C-3 olefin in compound **2** was found to be *Z* on the basis of 2D NOESY correlations between H_2_-1/H_2_-4 (δ_H_ 2.17) and H-3/H_3_-15. This assignment was further confirmed through 1D NOE experiments in which H_2_-1 and H_3_-15 were separately irradiated and found to display proximity through space to H_2_-4 and H-3, respectively. This proposed structure for **2** differs from that of iso-α-sinensyl acetate isolated from the terrestrial *Lomatium mohavense* ssp. *longilobum* by Beauchamp et al. (2010) only in the appearance of the C-2/C-3 olefin in the *Z* configuration.^7^

**Table 2:** NMR Data for Crannenols B, C, and D (**2**, **3**, and **4**)^a^

	crannenol B (**2**)		crannenol C (**3**)		crannenol D (**4**)	
pos	δ_C_,^b^ type	δ_H_^c^ (*J* in Hz)	δ_C_,^b^ type	δ_H_^c^ (*J* in Hz)	δ_C_,^b^ type	δ_H_^c^ (*J* in Hz)
1	63.2, CH_2_	4.58, s	60.2, CH_2_	4.71, s	58.5, CH_2_	4.18, s
2	129.8, C		133.7, C		133.7, C	
3	130.5, CH	5.38, t	133.3, CH	5.68, t,(7.5)	133.9, CH	5.64, t (7.5)
4	26.3, CH_2_	2.17, q (7.99)	26.0, CH_2_	2.25, q (7.5)	26.0, CH_2_	2.24, q (7.5)
5	39.5, CH_2_	2.02, t (7.81)	39.1, CH_2_	2.06, t	39.1, CH_2_	2.06, t (7.5)
6	134.9, C		134.5, C		134.6, C	
7	122.9, CH	5.12, m	123.3, CH	5.13, t	123.4, CH	5.13, t
8	26.2, CH_2_	2.87, t (7.27)	26.3, CH_2_	2.87, t (7.3)	26.3, CH_2_	2.87, t (7.3)
9	129.5, CH	5.35, t	129.4, CH	5.35, t (7.6)	129.3, CH	5.34, t (7.5)
10	132.0, C		132.2, C		132.2, C	
11	133.6, CH	6.81, q (17.4, 10.2)	133.6, CH	6.80, dd (17.4, 10.8)	133.5, CH	6.80, dd (18.2, 10.9)
12	113.5, CH_2_	5.11, d (10.9)	113.7, CH_2_	5.11, d (10.9)	113.7, CH_2_	5.11, d (10.9)
		5.21, d (17.1)		5.22, d (16.7)		5.22, d (17.1)
13	16.0, CH_3_	1.64, s	16.0, CH_3_	1.65, s	16.1, CH_3_	1.64, s
14	19.7, CH_3_	1.83, s	19.7, CH_2_	1.82, s	19.7, CH_2_	1.82, s
15	21.4, CH_3_	1.75, s	65.9, CH_2_	4.10, s	67.3, CH_2_	4.64, s
1′	171.2,^d^ C		171.4, C		171.4, C	
2′	21.0, CH_3_	2.08, s	21.0, CH_3_	2.08, s	21.0, CH_3_	2.09, s

a CDCl_3_, ppm, multiplicity determined by HSQC.

b 150 MHz.

c 600 MHz.

d Chemical shift confirmed by HMBC.

Crannenols C (**3**) and D (**4**) were isolated as clear oils with spectroscopic data similar to that of crannenols A (**1**) and B (**2**). A molecular formula of C_17_H_26_O_3_ for both compounds **3** and **4** was established by HRESIMS corroborated by ^1^H and ^13^C NMR spectra (Table 2). Both **3** and **4** differed from **2** by the presence of a new singlet methylene, H_2_-15 and H_2_-1, respectively (δ_H_ 4.10 and 4.18, respectively), signal in the ^1^H NMR spectra. The presence of an alcohol at C-15 and C-1 in **3** and **4** was suggested by the chemical shift of these carbons (δ_C_ 65.9 and 58.5, respectively). The assignment of this portion of **3** was determined through long-range COSY correlations between the singlet hydroxy-bearing methylene H_2_-15 and singlet acetoxy-bearing methylene H_2_-1 (δ_H_ 4.71) and to the triplet methine H-3 (δ_H_ 5.68), as well as HMBC correlations from H_2_-15 to C-1 (δ_C_ 60.2), C-2 (δ_C_133.7), and C-3 (δ_C_ 133.3). The assignment of this portion of **4** was determined through COSY correlations between the equivalent H_2_-1 to the triplet methine H-3 (δ_H_ 5.64) and the quartet methylene H_2_-4 (δ_H_ 2.24). Additionally, HMBC correlations from H_2_-1 to C-15 (δ_C_ 67.3), C-2 (δ_C_ 133.7), and C-3 (δ_C_ 133.9) confirmed this portion of the structure. The remainder of the carbon skeletons of **3** and **4** were found to mirror those of compounds **1** and **2** on the basis of ^1^H and ^13^C NMR data. The configuration of the C-2/C-3 olefin in **3** was found to be *Z* on the basis of 2D NOESY correlations between H_2_-1/H_2_-4 and H-3/H_2_-15. The configuration of the C-2/C-3 olefin in **4** was found to be *E* on the basis of 2D NOESY correlations of H_2_-15/H-3 and H_2_-4/H_2_-1.

Due to the abundance of crannenol A (**1**) isolated, this metabolite was used as a probe for the evaluation of biological activity of the series. Despite the observed activity of the organic extracts, analyses of purified compound **1** revealed no discernible antibiotic activity, based on screening seven strains of *Candida* spp., the ESKAPE pathogens, *Mycobacterium* tuberculosis, and human respiratory syncytial virus.

Despite the lack of retention of biological activity from extract to metabolite, this study reports the isolation and elucidation of a series of four undescribed compounds, crannenols A–D (**1**–**4**), from a genus of deep-sea soft coral for which no chemical investigations have been reported.

## Experimental Section

### General Experimental Procedures

Solvents were obtained from Fisher Scientific Co. and were HPLC grade (>99% purity) unless otherwise stated. UV absorptions were measured with a Shimadzu LC-20AT HPLC system equipped with a Shimadzu SPD-M20A diode array detector in CH_3_OH. IR spectra were recorded with an Agilent Cary 630 FTIR. NMR spectra were acquired in CDCl_3_ with residual solvent referenced as the internal standard (δ_H_ 7.27; δ_C_ 77.0) for ^1^H and ^13^C NMR spectra, respectively. The NMR spectra were recorded on a Varian 600 MHz broadband instrument operating at 600 MHz for ^1^H and 150 MHz for ^13^C. GC/MS analysis was performed on an Agilent 7890A GC using a Zebron ZB-5HT Inferno (30 m × 0.25 mm, 0.25 μm film thickness) column coupled to an Agilent 7200 accurate-mass QToF with electron impact ionization. LC/MS analysis was performed on an Agilent 1260 Infinity LC using an analytical C18 (150 × 3.0 mm, 2.6 μm) column coupled to an Agilent 6540 UHD accurate-mass QToF with electrospray ionization. MPLC fractionation and analysis were performed on a Teledyne-Isco CombiFlash Rf system equipped with built-in UV detection at 254 and 280 nm. HPLC fractionation and analysis were performed on a Shimadzu LC-20AR system equipped with a Shimadzu SPD-20A UV/vis detector using preparative silica or semipreparative C18 ((250 × 21.2 mm, 5 μm) or (250 × 10.0 mm, 5 μm)) conditions.

### Biological Materials

Thirty-one specimens of *Acanella arbuscula* (Cnidaria, Alcyonacea, Calcaxonia, Keratoisididae) were collected from the Whittard Canyon, an extensive submarine canyon system southwest of Ireland on the northeast Atlantic margin, between May 30 and June 10, 2016, using the ROV *Holland I* deployed from the Irish national research vessel RV *Celtic Explorer*. Specimens were collected from depths of 984–2011 m during a series of eight ROV dives that ranged in latitude from 48° 25′ 42″ N to 48° 40′ 23″ N and in longitude from 9° 52′ 58″ W to 10° 40′ 50″ W. Specimens were stored in bioboxes on the ROV and immediately identified, logged, labeled, and frozen at −80 °C when the ROV was recovered to the vessel. Specimens were freeze-dried on return to land and then stored until analysis at −20 °C. Specimens were identified as *A. arbuscula* based on a distinctive skeleton of alternating proteinaceous nodes with calcium carbonate internodes and the densely branched, bush-like structure of the colonies.

According to the latest taxonomic revision, *A*. *arbuscula* displays widely divergent morphotypes within this general morphology but is the only species of *Acanella* present in the northeast Atlantic.^8^

### Extraction and Isolation

Crannenols A, C, and D (**1**, **3**, **4**) were isolated from 720.5 g of 31 combined freeze-dried *A*. *arbuscula* specimens extracted via Soxhlet extraction in CHCl_2_ and dried in vacuo, resulting in 5.9 g of organic extract. The extract was fractionated using MPLC utilizing a gradient from 100% hexanes to 100% EtOAc with a normal phase silica column over 25 min, resulting in eight fractions following the recombination of fractions with similar UV profiles. Fraction D/E (320 mg) was shown by ^1^H NMR spectroscopy to contain a chemical shift pattern consistent with terpene-like secondary metabolites and was thus subjected to normal phase HPLC utilizing a gradient from 100% hexanes to 60% EtOAc over 25 min on a preparative silica column, affording crannenol A (**1**, 35.6 mg). In MPLC fraction F a doublet of doublets at a chemical shift of 6.80 ppm was observed, mirroring that seen in compound **1** as the β-vinylic protons on C-11. Further HPLC separation of this fraction was conducted on a preparative silica column with a gradient from 78% hexanes to 53% EtOAc over 17 min to yield two terpene-containing fractions, 7 and 8. Each of these fractions was individually subjected to reversed phase HPLC on a semipreparative C18 column with a gradient from 75% to 100% MeOH over 17 min to afford crannenols C (**3**, 2.2 mg) and D (**4**, 1.8 mg), respectively.

Crannenol B (**2**) was isolated from subsequent Soxhlet extraction in MeOH of the same 31 combined freeze-dried *A*. *arbuscula* specimens following CHCl_2_ extraction and dried in vacuo, resulting in 35.6 g of organic extract. The extract was fractionated using MPLC utilizing a solvent system of 5% MeOH with a reversed phase C18 column for 10 min to elute the majority of highly polar compounds followed by an immediate increase to 100% MeOH over 0.1 min that was held for 15 min to elute less polar secondary metabolites, resulting in two fractions. ^1^H NMR analysis of the nonpolar fraction 2 confirmed the presence of a similar analog to that of crannenols A, C, and D (**1**, **3**, **4**) and was thus subjected to normal phase HPLC utilizing a gradient from 100% hexanes to 70% EtOAc over 14 min and shown to contain both crannenol A (**1**) in the resulting fraction 2 and a separate analog in fraction 1. Fraction 1 was further subjected to reversed phase HPLC on an analytical C18 column with a gradient from 50% to 100% MeOH over 20 min, affording crannenol B (**2**, 0.1 mg).

#### Crannenol A (**1**):

clear oil; UV (MeOH) λ_max_ 236 nm; IR ν (thin film) 2943, 1744, 1446, 1379, 1223, 1029, 970, 910, 612 cm^–1^; ^1^H and ^13^C NMR data, Table 1; 70 eV HREIMS *m*/*z* 320.1984 [M]^+^ (calcd for C_19_H_28_O_4_, 320.1982)

#### Crannenol B (**2**):

clear oil; UV (MeOH) λ_max_ 236 nm; IR ν (thin film) 2935, 1744, 1454, 1379, 1245, 1037, 992 cm^–1^; ^1^H and ^13^C NMR data, Table 2; 70 eV HREIMS *m*/*z* 262.1922 [M]^+^ (calcd for C_17_H_26_O_2_, 262.1927).

#### Crannenol C (**3**):

clear oil; UV (MeOH) λ_max_ 235 nm; IR ν (thin film) 3441, 2943, 1744, 1454, 1379, 1245, 1037 cm^–1^; ^1^H and ^13^C NMR data, Table 2; HRESIMS *m*/z 279.1955 [M + H]^+^ (calcd for C_17_H_26_O_3_, 279.1955).

#### Crannenol D (**4**):

clear oil; UV (MeOH) λ_max_ 235 nm; IR ν (thin film) 3449, 2935, 1744, 1446, 1387, 1238, 1029 cm^–1^; ^1^H and ^13^C NMR data, Table 2; HRESIMS *m*/*z* 279.1953 [M + H]^+^ (calcd for C_17_H_26_O_3_, 279.1955).

## Supporting Information Available

NMR spectra of crannenols A–D (**1**–**4**); HREIMS of **1** and **2**; HRESIMS of **3** and **4**; UV λ_max_ of **1**–**4**; IR spectra of **1**–**4** (PDF)

## References

[ref1] Newman D. J.; Cragg G. M. J. Nat. Prod 2020, 83, 770–803. 10.1021/acs.jnatprod.9b01285. 32162523

[ref2] Skropeta D. Nat. Prod. Rep 2008, 25, 1131–1166. 10.1039/b808743a. 19030606

[ref3] Pilkington L. I. Molecules 2019, 24, 1–24. 10.3390/molecules24213942. PMC6865307 31683674

[ref4] Kobayashi M.; Yasuzawa T.; Kobayashi Y.; Kyogoku Y.; Kitagawa I. Tetrahedron Lett. 1981, 22, 4445–4448. 10.1016/S0040-4039(01)82979-8.

[ref5] Roy P. K; Maarisit W.; Roy M. C; Taira J.; Ueda K. Mar. Drugs 2012, 10, 2741–2748. 10.3390/md10122741. 23201595 PMC3528123

[ref6] Palermo J. A; Brasco M. F; Spagnuolo C.; Seldes A. M. J. Org. Chem. 2000, 65, 4482–4486. 10.1021/jo991740x. 10959848

[ref7] Beauchamp P. S; Dev V.; Kittisanthanon K.; Ly B. Flavour Fragr. J. 2010, 25, 427–430. 10.1002/ffj.1990.

[ref8] Saucier E. H; Sajjadi A.; France S. C. Zootaxa 2017, 4323 (3), 359–390. 10.11646/zootaxa.4323.3.2.

