# Supplementary material for: Crannenols A–D, Sesquiterpenoids from the Irish Deep-Sea Soft Coral Acanella arbuscula
Source: J Nat Prod. 2022 Sep 19;85(10):2395–8. doi: 10.1021/acs.jnatprod.2c00602 (PMC9623587; doi:10.1021/acs.jnatprod.2c00602)
Supplement: Supplementary file 1 — np2c00602_si_001.pdf [file np2c00602_si_001.pdf]

Supplemental Information for:

Crannenols A-D, Sesquiterpenoids from the Irish Deep-Sea Soft Coral *Acanella arbuscula*

Joshua T. Welsch,[†] *Ryan M. Young,*[‡] *A. Louise Allcock,*[‡] *Mark P. Johnson,*[‡] *Bill J. Baker*[†]

[†]Department of Chemistry, University of South Florida, Tampa, FL, USA.

[‡]School of Natural Sciences and Ryan Institute, University of Galway, University Road, Galway, H91 TK33, Ireland

.

	Page
Figure S1: Crannenol A (**1**) ^{1}H NMR spectrum (600 MHz, $CDCl_3$).	2
Figure S2: Crannenol A (**1**) ^{13}C NMR spectrum (150 MHz, $CDCl_3$).	3
Figure S3: Crannenol A (**1**) HSQC NMR spectrum (500 MHz, $CDCl_3$).	3
Figure S4: Crannenol A (**1**) HMBC NMR spectrum (600 MHz, $CDCl_3$).	4
Figure S5: Crannenol A (**1**) COSY NMR spectrum (600 MHz, $CDCl_3$).	4
Figure S6: Crannenol A (**1**) NOESY NMR spectrum (600 MHz, $CDCl_3$).	5
Figure S7: Crannenol A (**1**) HREIMS.	5
Figure S8: Crannenol A (**1**) UV λ_{max} (CH_3OH).	6
Figure S9: Crannenol A (**1**) IR spectrum (thin film).	6
Figure S10: Crannenol B (**2**) ^{1}H NMR spectrum (600 MHz, $CDCl_3$).	7
Figure S11: Crannenol B (**2**) ^{13}C NMR spectrum (150 MHz, $CDCl_3$).	7
Figure S12: Crannenol B (**2**) ^{13}C NMR spectrum zoomed (150 MHz, $CDCl_3$).	8
Figure S13: Crannenol B (**2**) DEPT135 NMR spectrum (600 MHz, CDCl3).	8
Figure S14: Crannenol B (**2**) HSQC NMR spectrum (600 MHz, $CDCl_3$).	9
Figure S15: Crannenol B (**2**) HMBC NMR spectrum (500 MHz, $CDCl_3$).	9
Figure S16: Crannenol B (**2**) COSY NMR spectrum (600 MHz, $CDCl_3$).	10
Figure S17: Crannenol B (**2**) NOESY NMR spectrum (500 MHz, $CDCl_3$).	10
Figure S18: Crannenol B (**2**) 1D NOE irradiating protons at 4.58ppm (400 MHz, $CDCl_3$).	11
Figure S19: Crannenol B (**2**) 1D NOE irradiating protons at 1.75ppm (400 MHz, $CDCl_3$).	11
Figure S20: Crannenol B (**2**) HREIMS.	12

Figure S21: Crannenol B (**2**) UV λ_{max} (CH_3OH). 12
Figure S22: Crannenol B (**2**) IR spectrum (thin film). 13
Figure S23: Crannenol C (**3**) ^{1}H NMR spectrum (600 MHz, $CDCl_3$). 13
Figure S24: Crannenol C (**3**) ^{13}C NMR spectrum (150 MHz, $CDCl_3$). 14
Figure S25: Crannenol C (**3**) HSCQ NMR spectrum (500 MHz, $CDCl_3$). 14
Figure S26: Crannenol C (**3**) HMBC NMR spectrum (500 MHz, $CDCl_3$). 15
Figure S27: Crannenol C (**3**) COSY NMR spectrum (500 MHz, $CDCl_3$). 15
Figure S28: Crannenol C (**3**) NOESY NMR spectrum (500 MHz, $CDCl_3$). 16
Figure S29: Crannenol C (**3**) HRESIMS. 16
Figure S30: Crannenol C (**3**) UV λ_{max} (CH_3OH). 17
Figure S31: Crannenol C (**3**) IR spectrum (thin film). 17
Figure S32: Crannenol D (**4**) ^{1}H NMR spectrum (600 MHz, $CDCl_3$). 18
Figure S33: Crannenol D (**4**) ^{13}C NMR spectrum (150 MHz, $CDCl_3$). 18
Figure S34: Crannenol D (**4**) HSCQ NMR spectrum (500 MHz, $CDCl_3$). 19
Figure S35: Crannenol D (**4**) HMBC NMR spectrum (500 MHz, $CDCl_3$) 19
Figure S36: Crannenol D (**4**) COSY NMR spectrum (600 MHz, $CDCl_3$). 20
Figure S37: Crannenol D (**4**) NOESY NMR spectrum (500 MHz, $CDCl_3$). 20
Figure S38: Crannenol D (**4**) HRESIMS. 21
Figure S39: Crannenol D (**4**) UV λ_{max} (CH_3OH). 21
Figure S40: Crannenol D (**4**) IR spectrum (thin film). 22

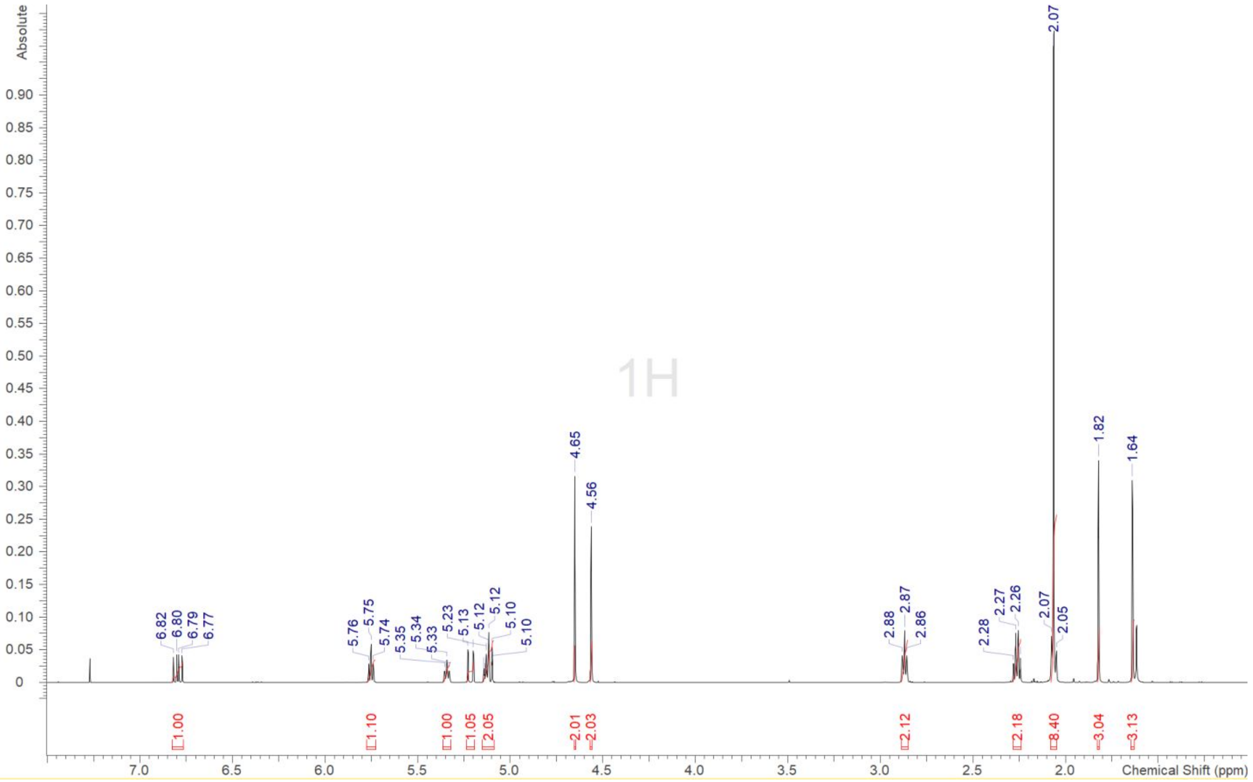

Figure S1: Crannenol A (**1**) ^{1}H NMR spectrum (600 MHz, $CDCl_3$).

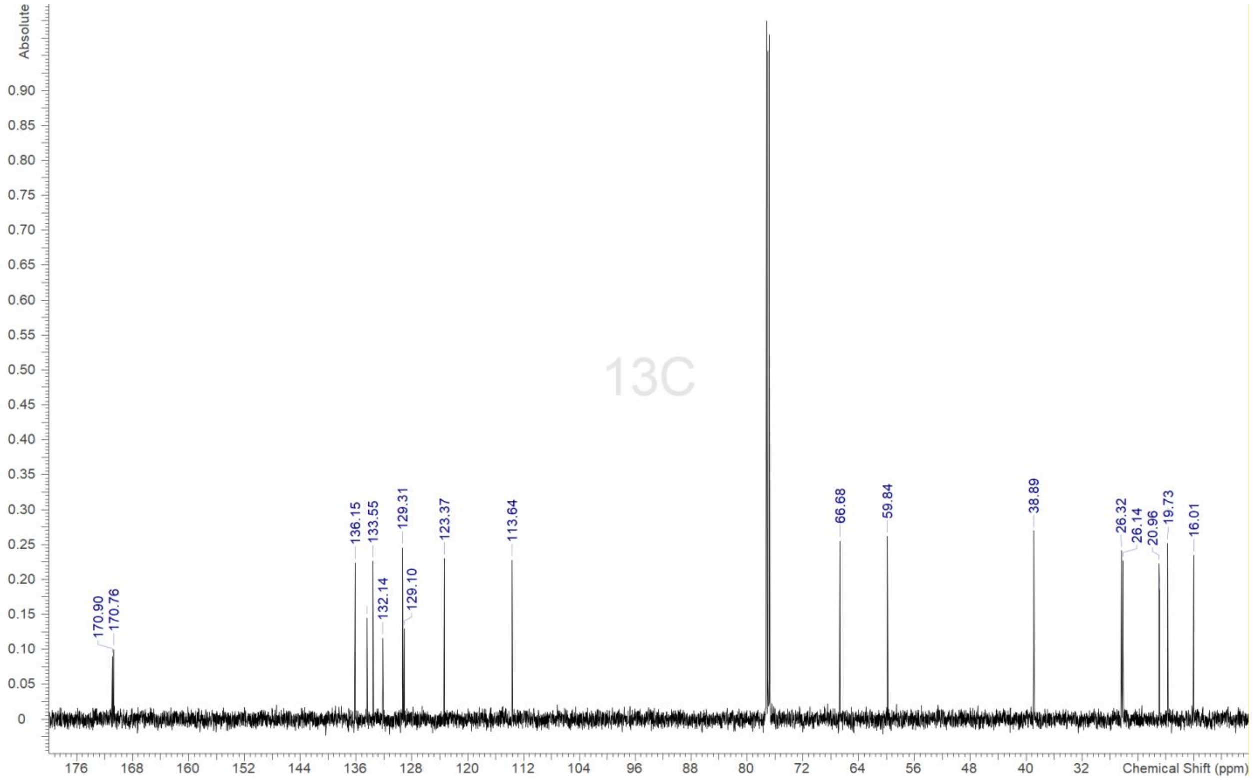

Figure S2: Crannenol A (**1**) ^{13}C NMR spectrum (150 MHz, $CDCl_3$).

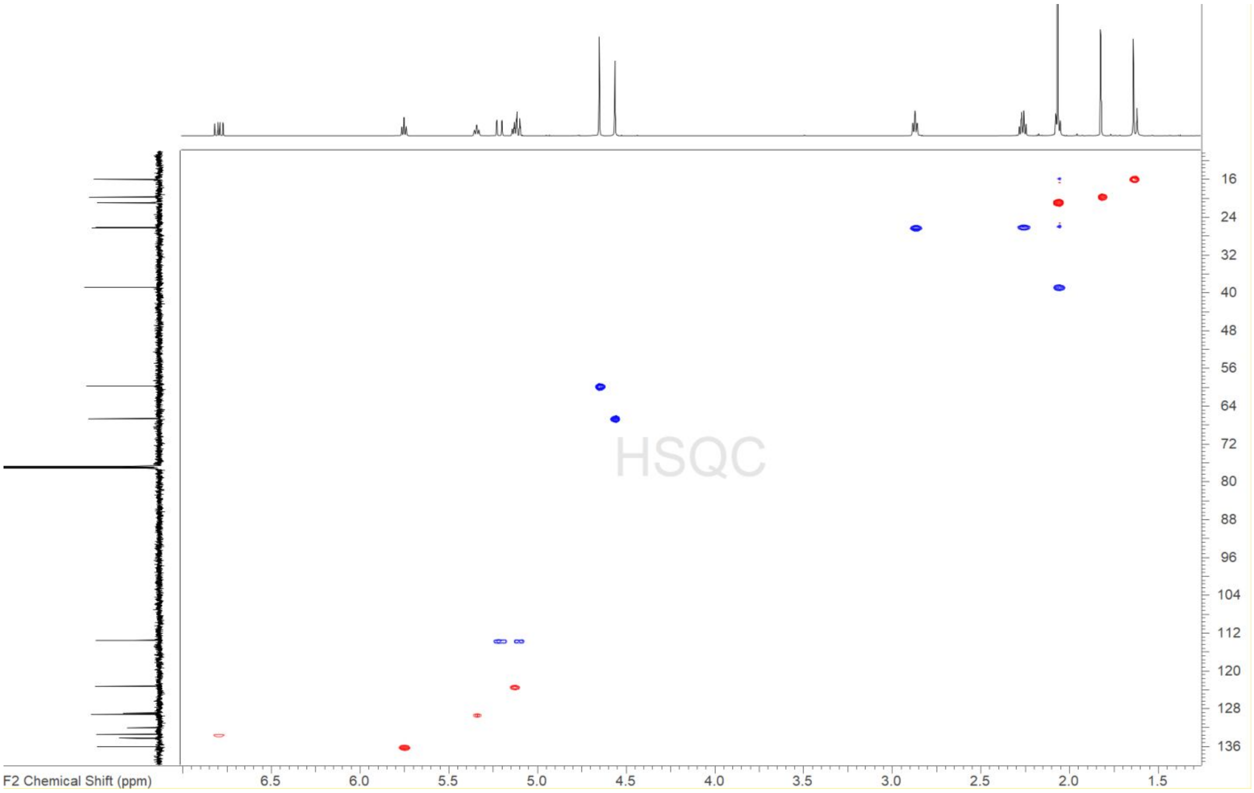

Figure S3: Crannenol A (**1**) HSQC spectrum (500 MHz, $CDCl_3$).

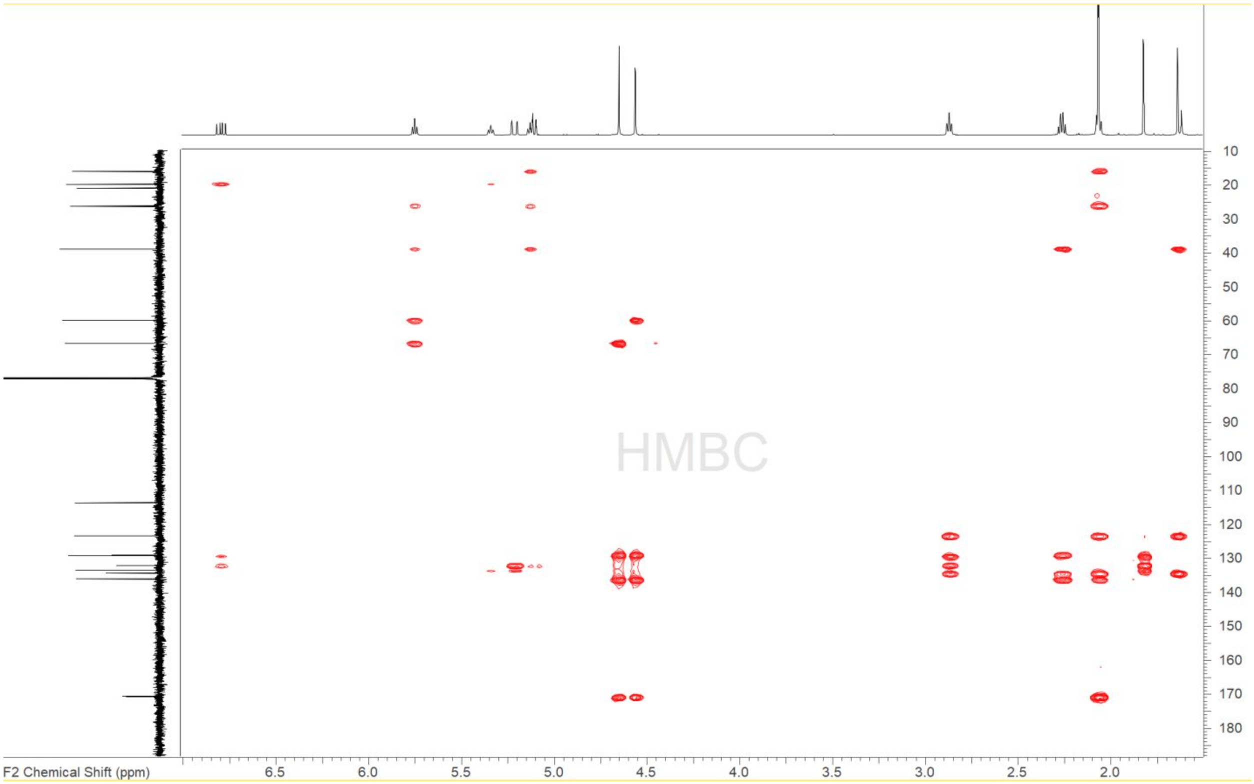

Figure S4: Crannenol A (**1**) HMBC spectrum (600 MHz, $CDCl_3$).

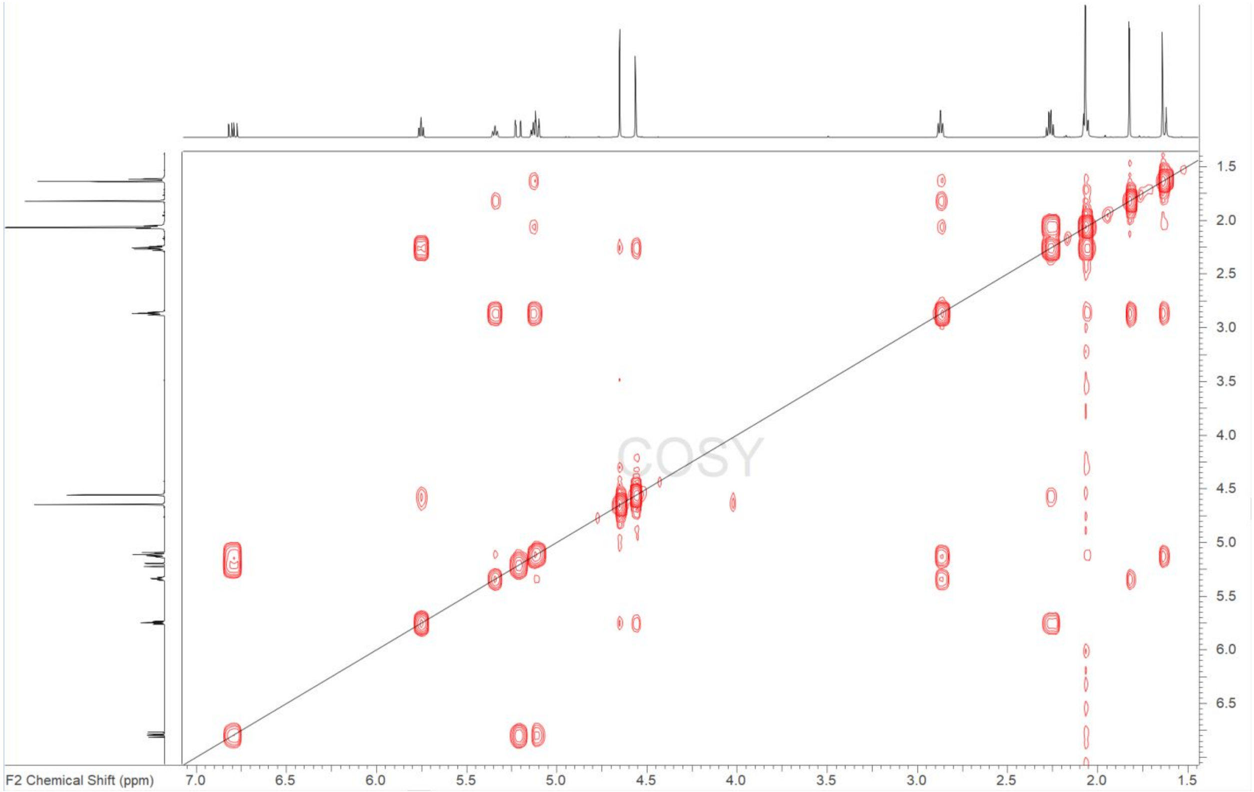

Figure S5: Crannenol A (**1**) COSY spectrum (600 MHz, $CDCl_3$).

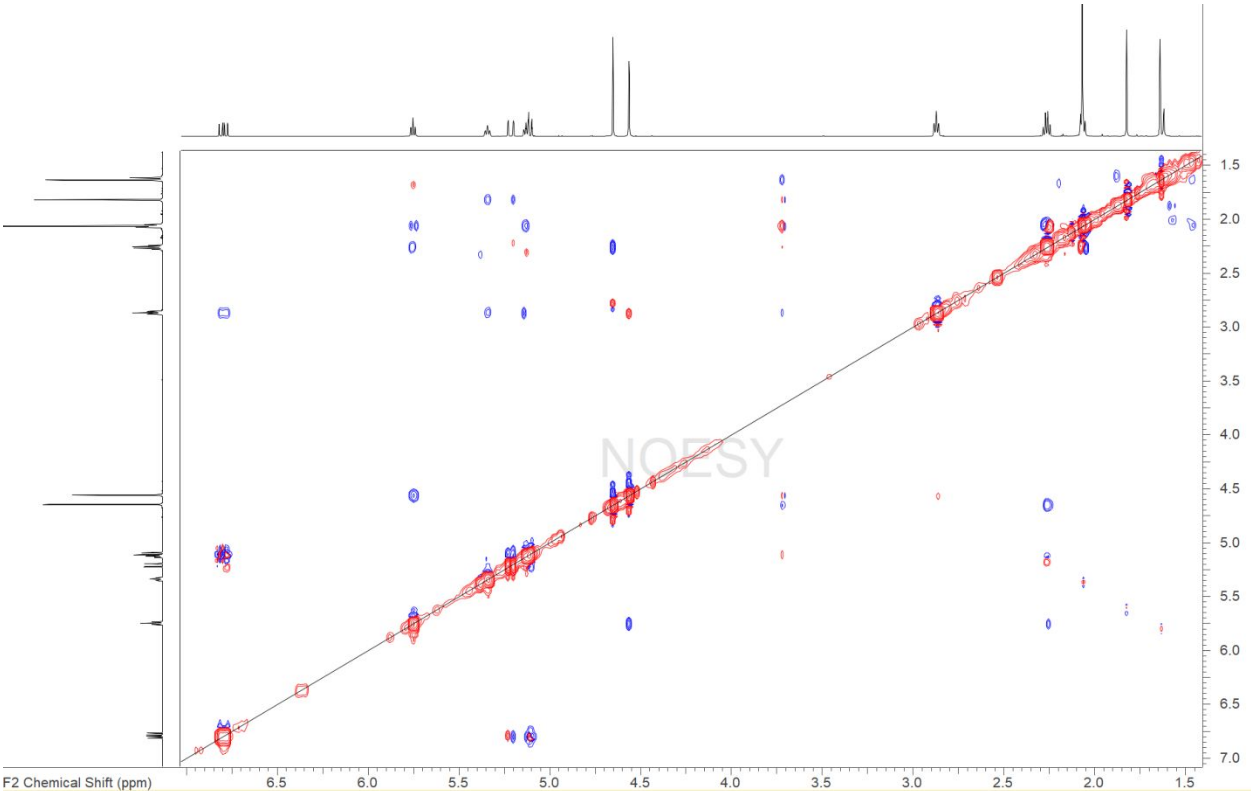

Figure S6: Crannenol A (**1**) NOESY spectrum (600 MHz, $CDCl_3$).

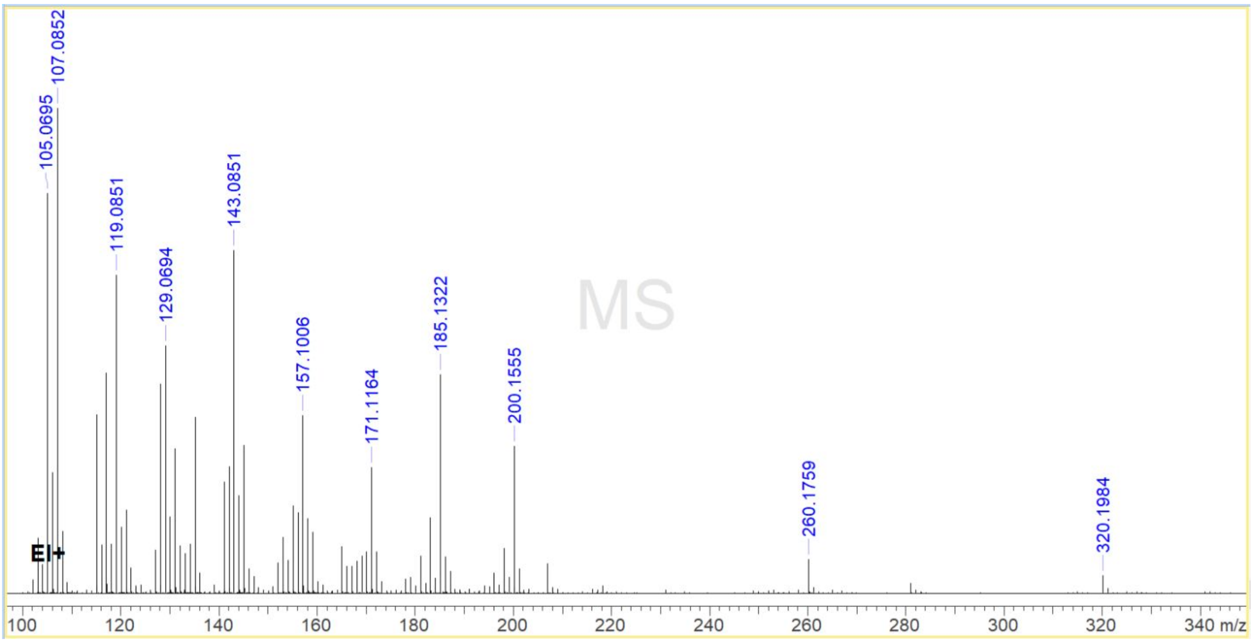

Figure S7: Crannenol A (**1**) HREIMS.

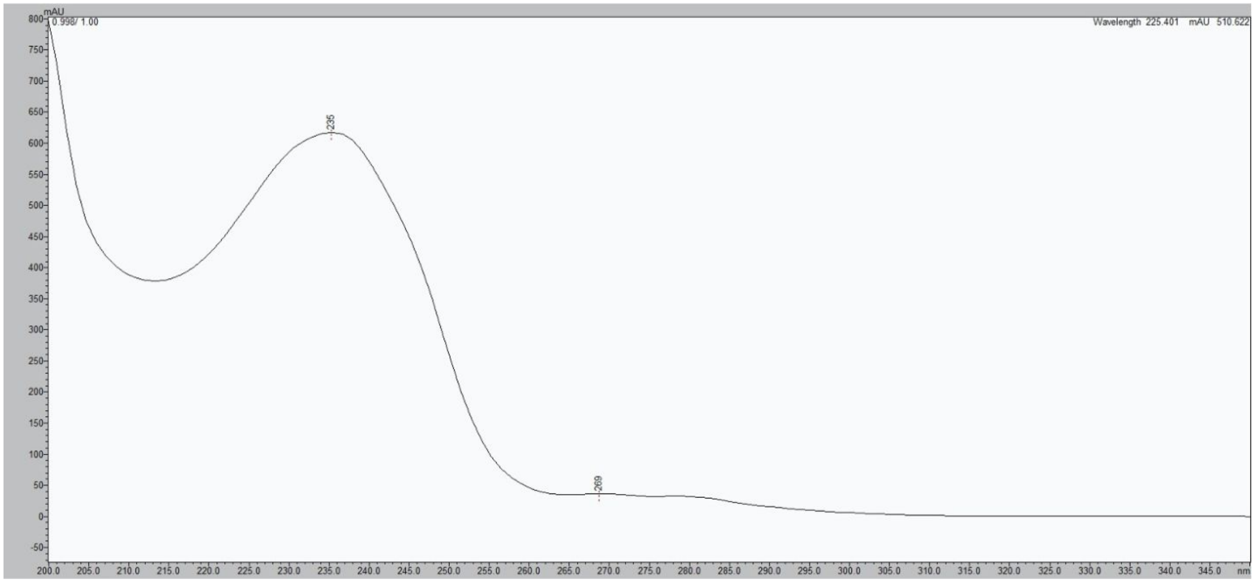

Figure S8: Crannenol A (**1**) UV λ_{max} (CH_3OH).

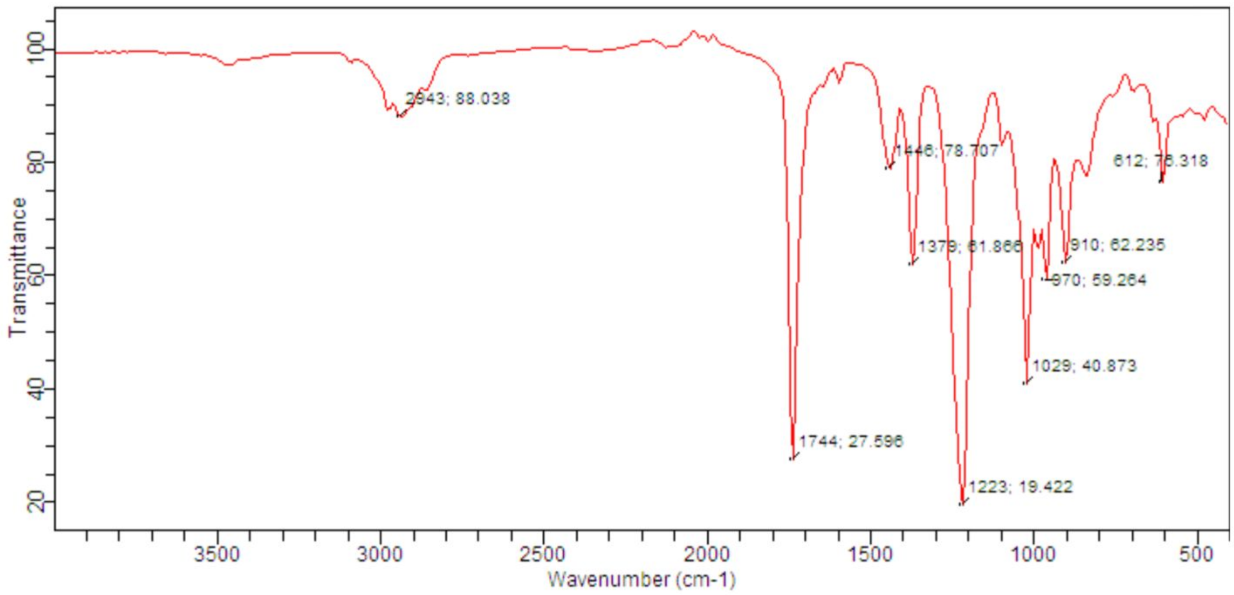

Figure S9: Crannenol A (**1**) IR spectrum (thin film).

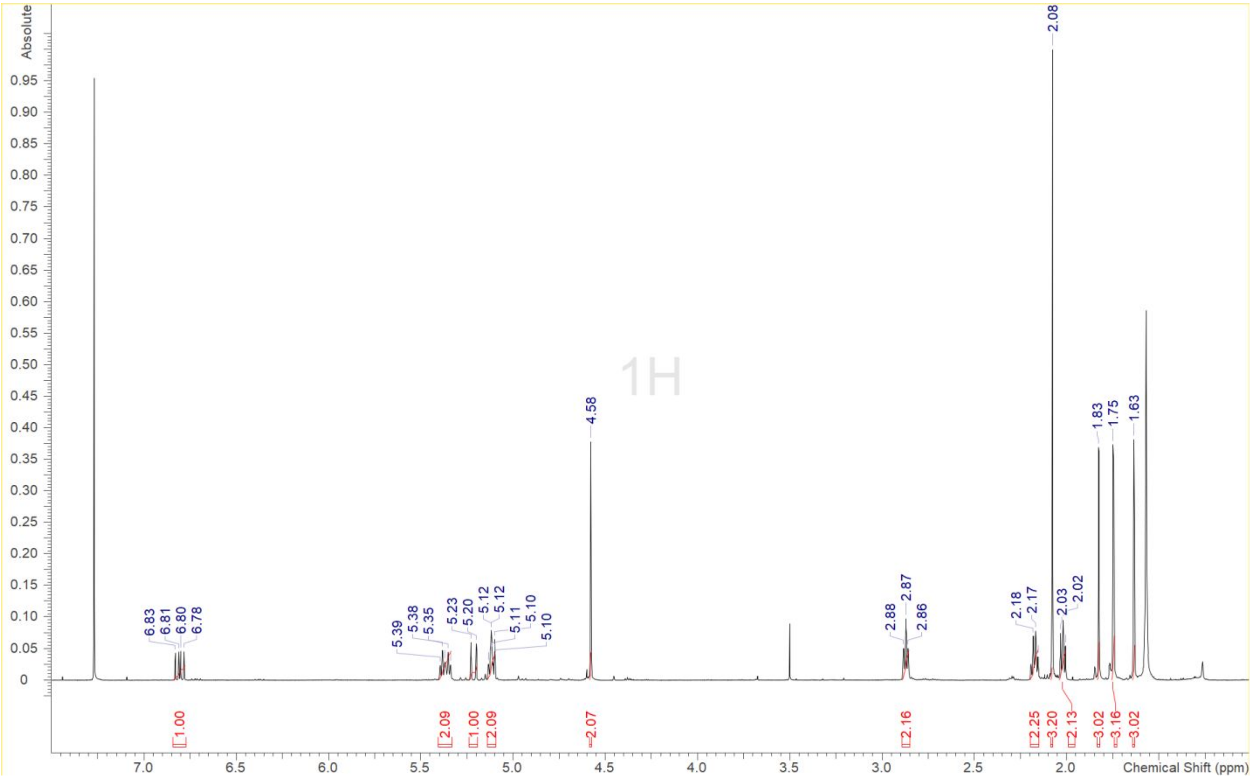

Figure S10: Crannenol B (**2**) ^{1}H NMR spectrum (600 MHz, $CDCl_3$).

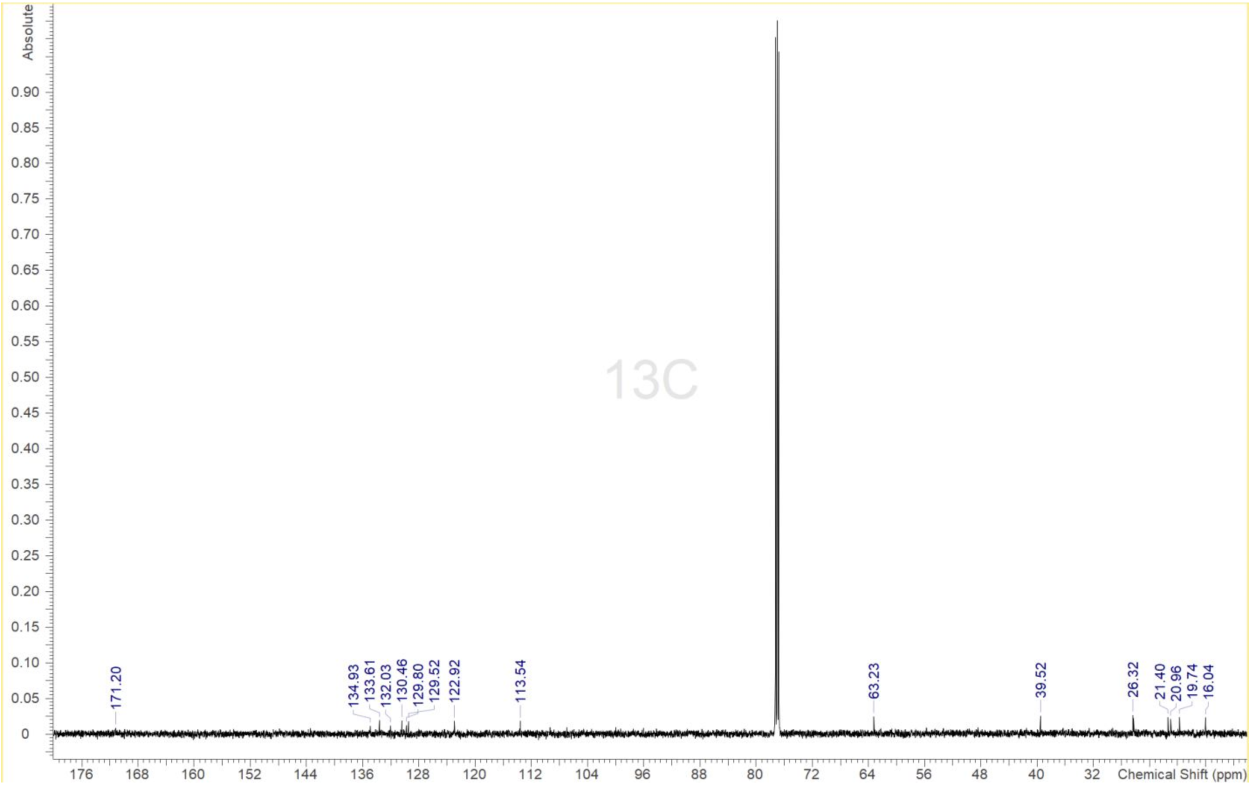

Figure S11: Crannenol B (**2**) ^{13}C NMR spectrum (150 MHz, $CDCl_3$).

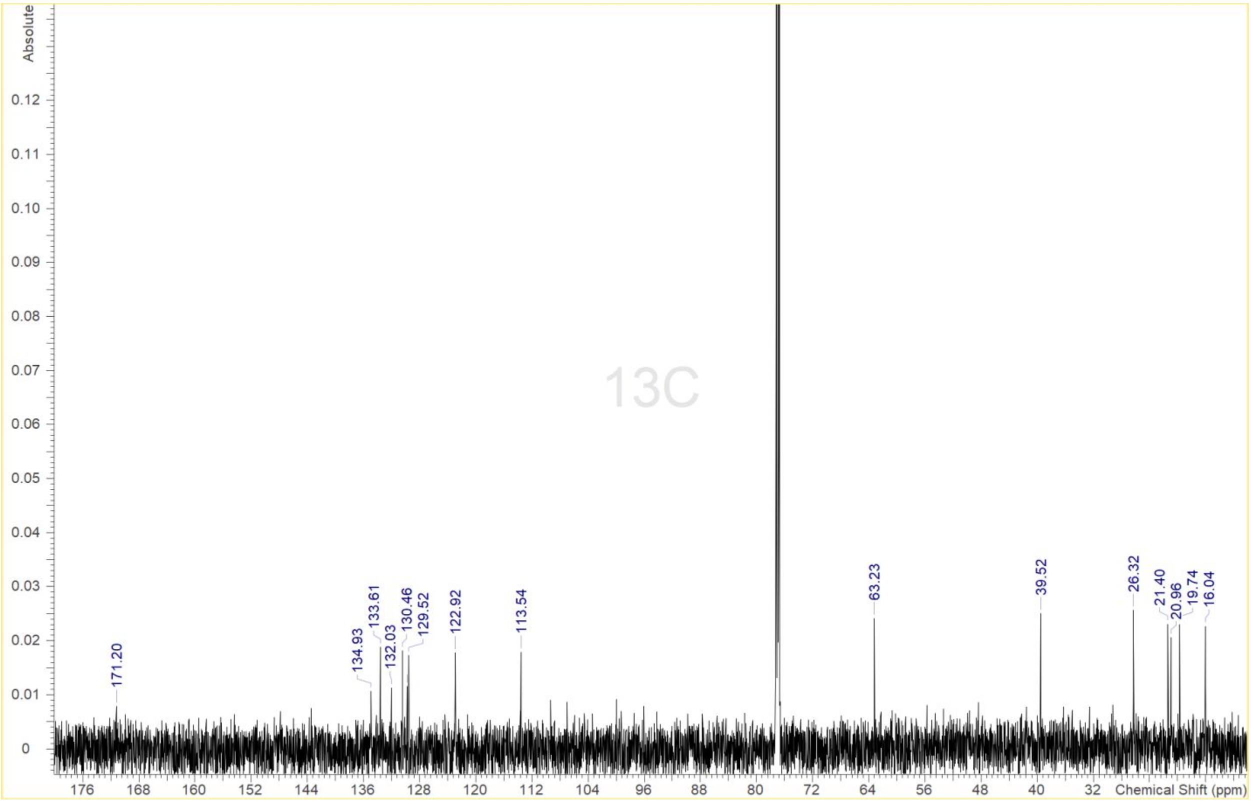

Figure S12: Crannenol B (**2**) ^{13}C NMR spectrum zoomed (150 MHz, $CDCl_3$).

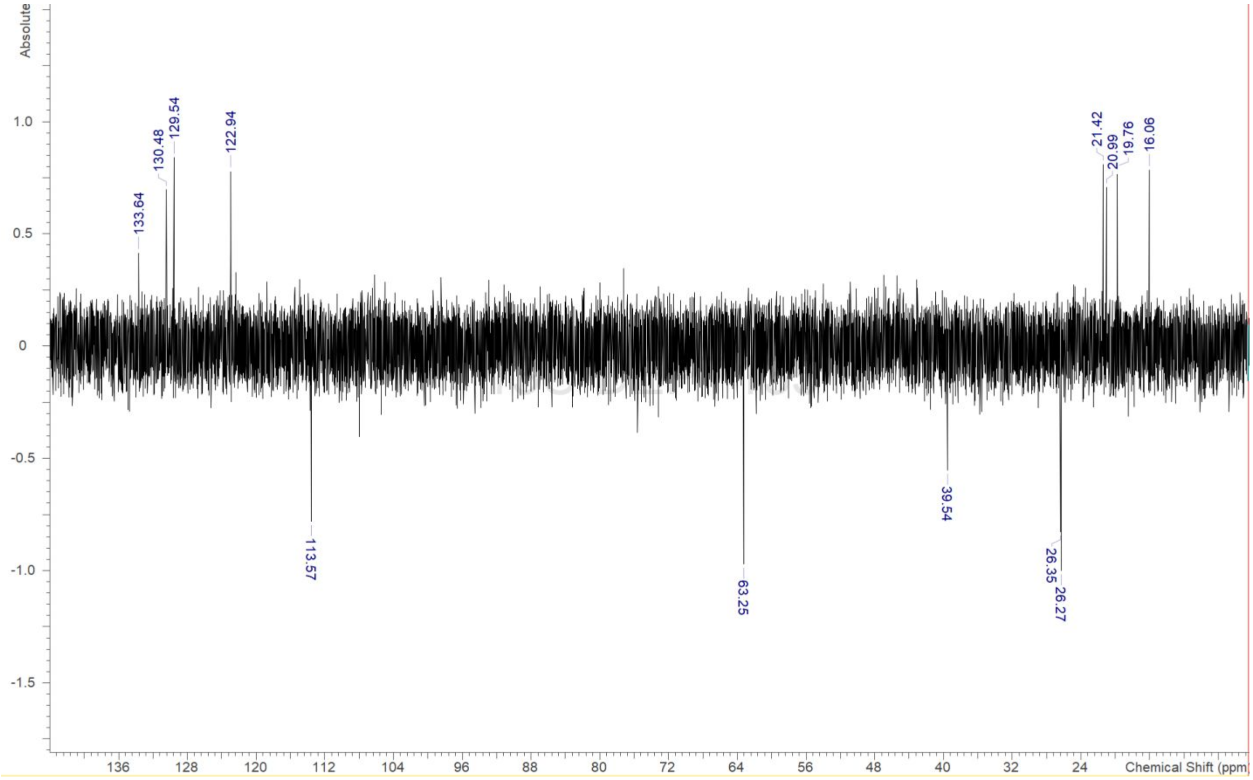

Figure S13: Crannenol B (**2**) DEPT135 NMR spectrum (150 MHz, $CDCl_3$).

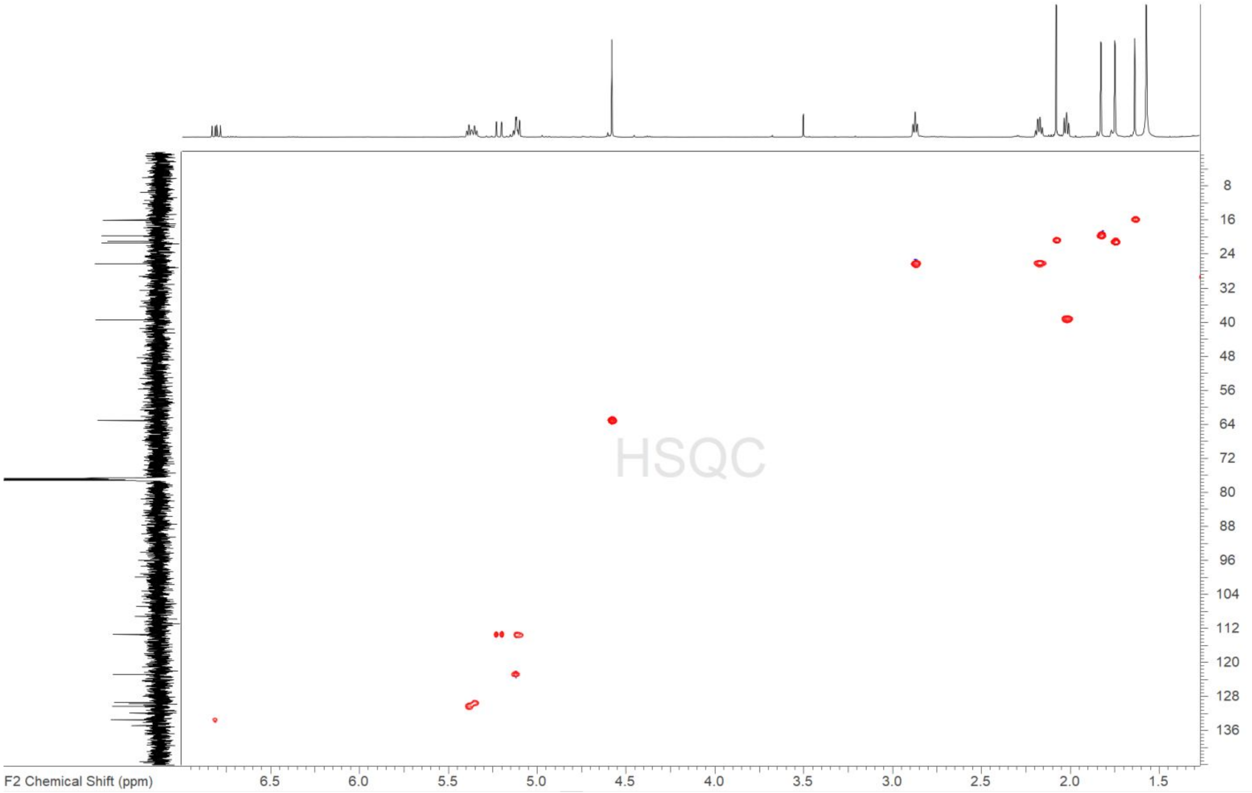

Figure S14: Crannenol B (**2**) HSQC NMR spectrum (600 MHz, $CDCl_3$).

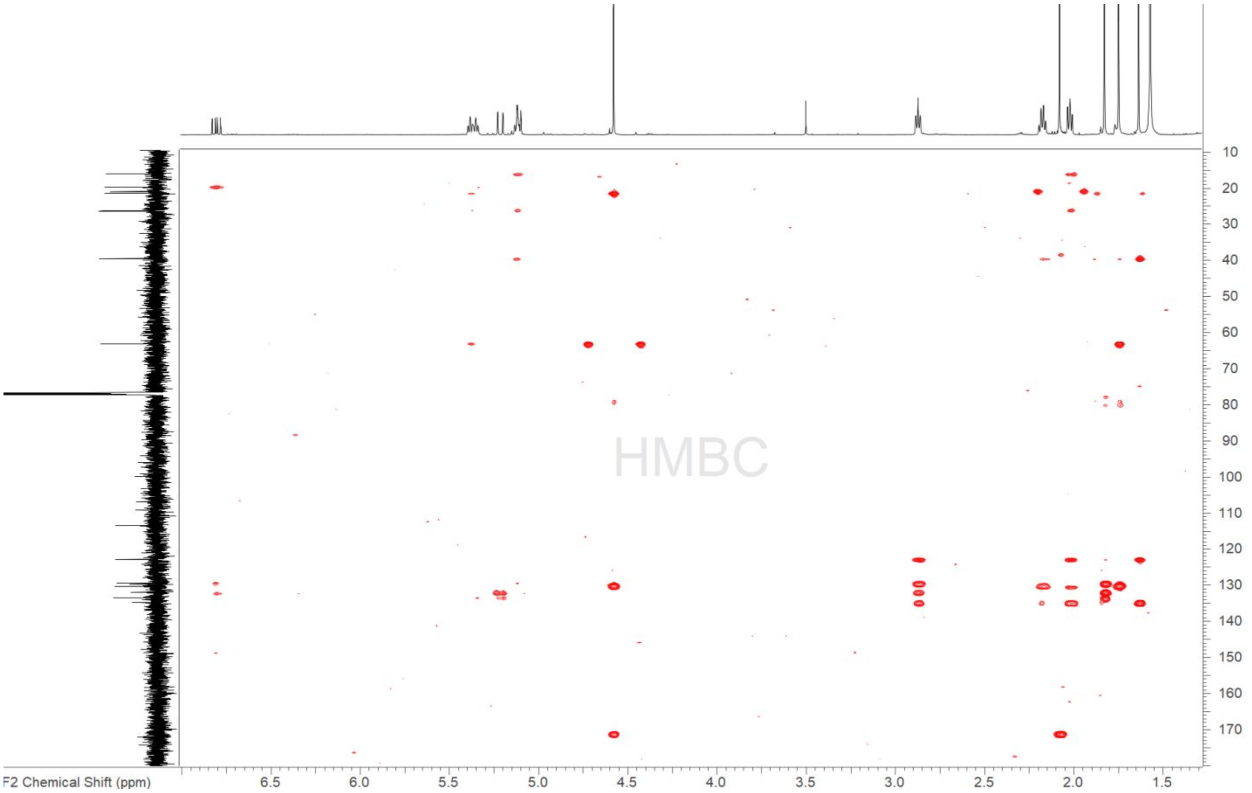

Figure S15: Crannenol B (**2**) HMBC NMR spectrum (500 MHz, $CDCl_3$).

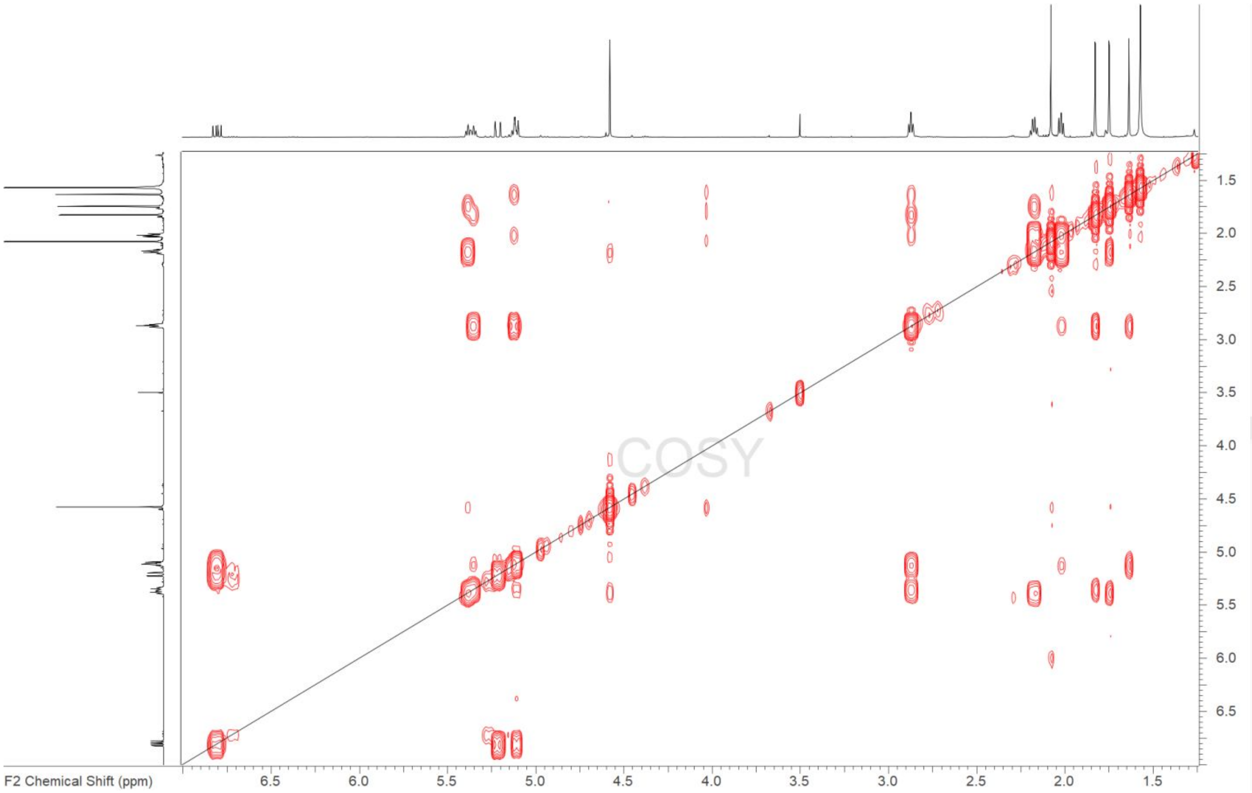

Figure S16: Crannenol B (**2**) COSY NMR spectrum (600 MHz, $CDCl_3$).

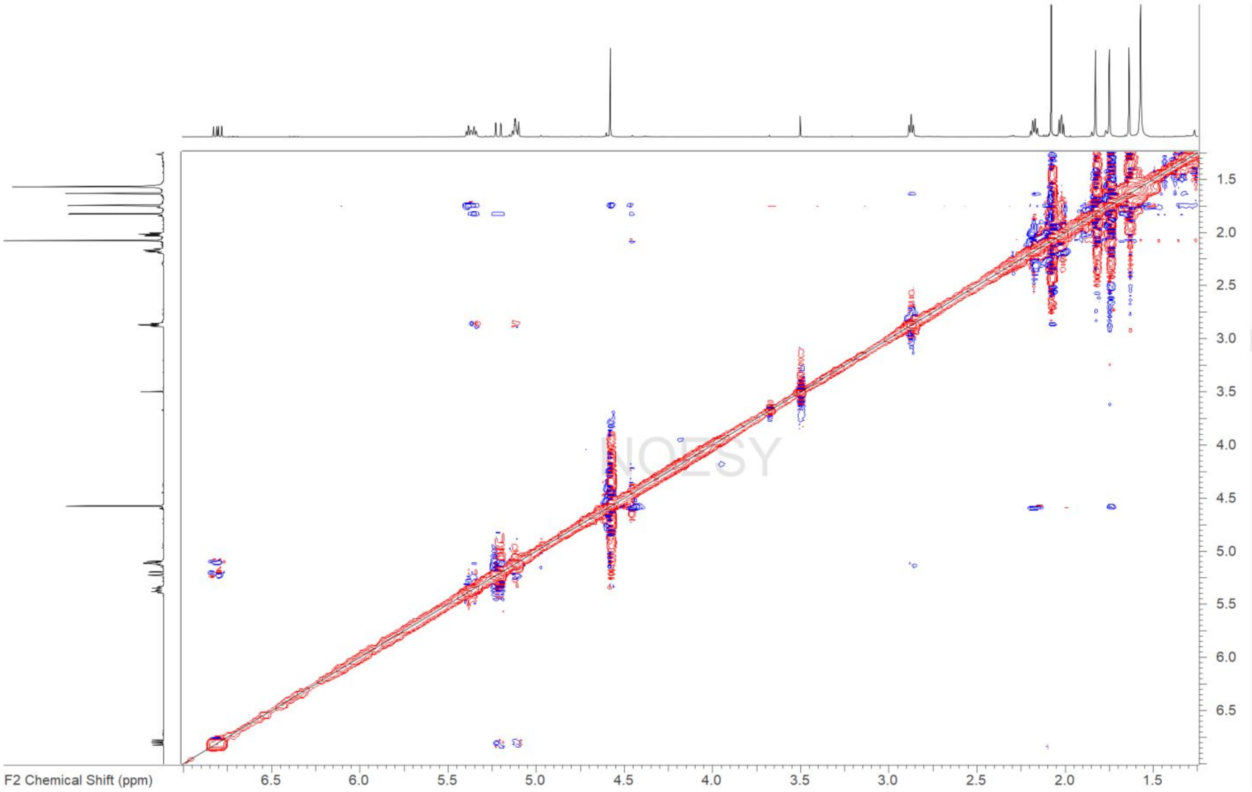

Figure S17: Crannenol B (**2**) NOESY NMR spectrum (500 MHz, $CDCl_3$).

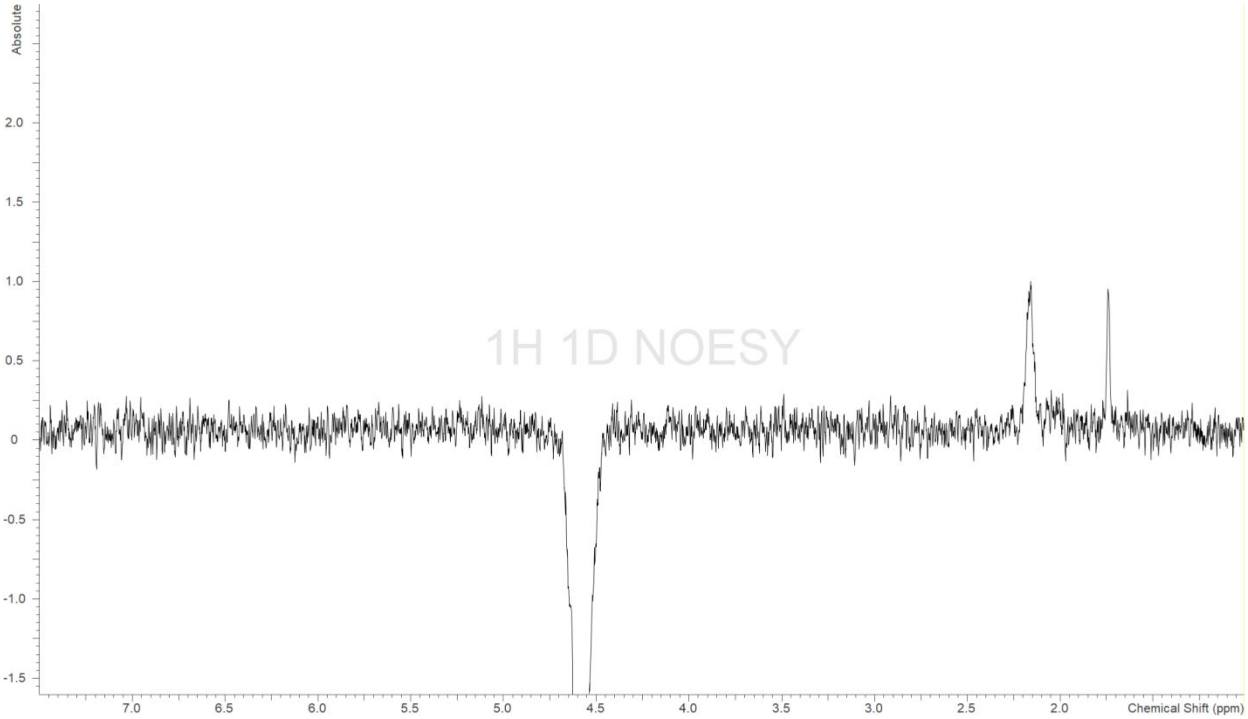

Figure S18: Crannenol B (**2**) 1D NOE NMR spectrum irradiating protons at 4.58ppm (400 MHz, $CDCl_3$).

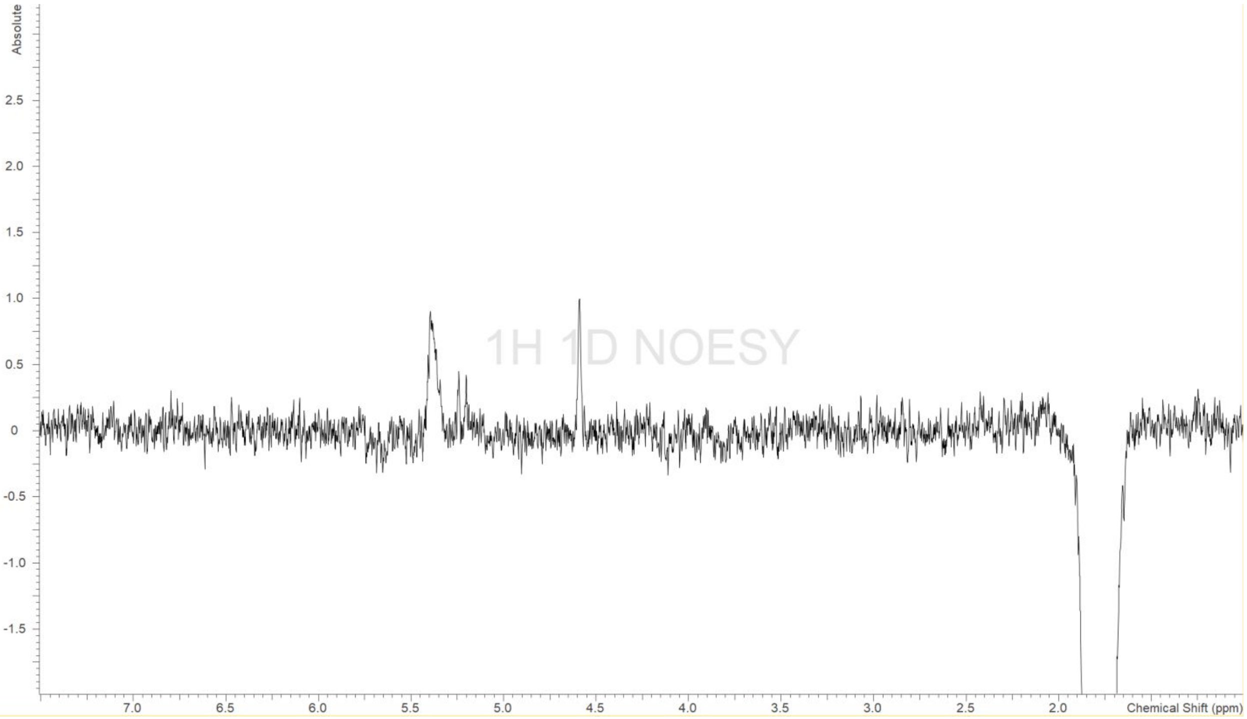

Figure S19: Crannenol B (**2**) 1D NOE NMR spectrum irradiating protons at 1.75ppm (400 MHz, $CDCl_3$).

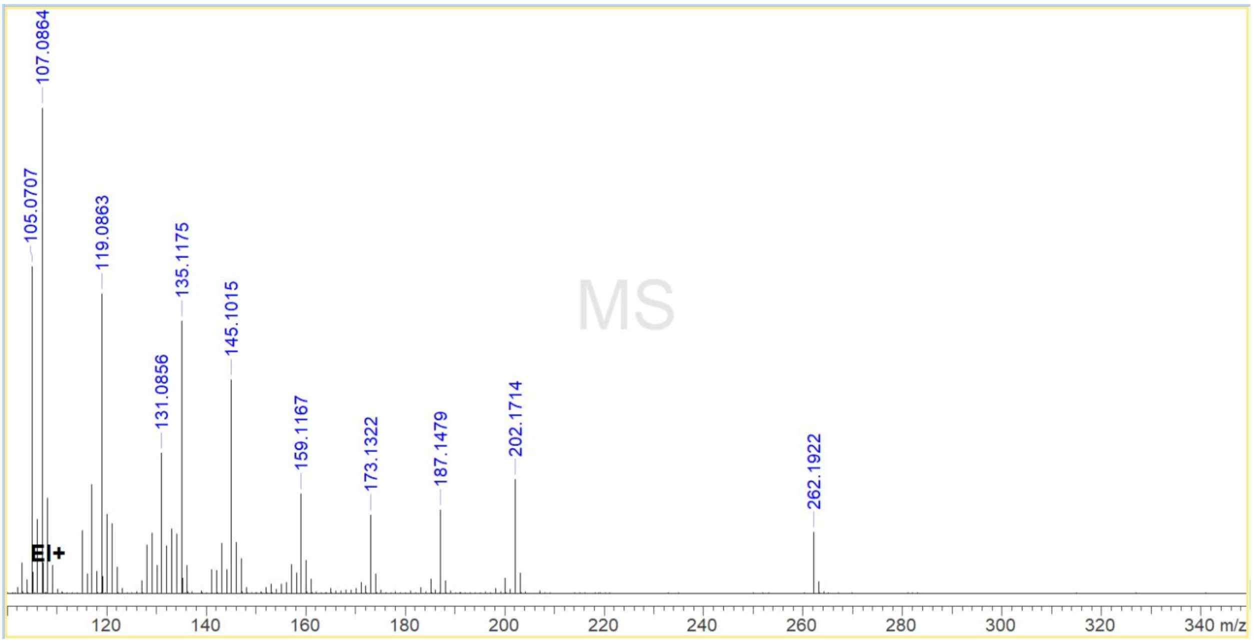

Figure S20: Crannenol B (**2**) HREIMS.

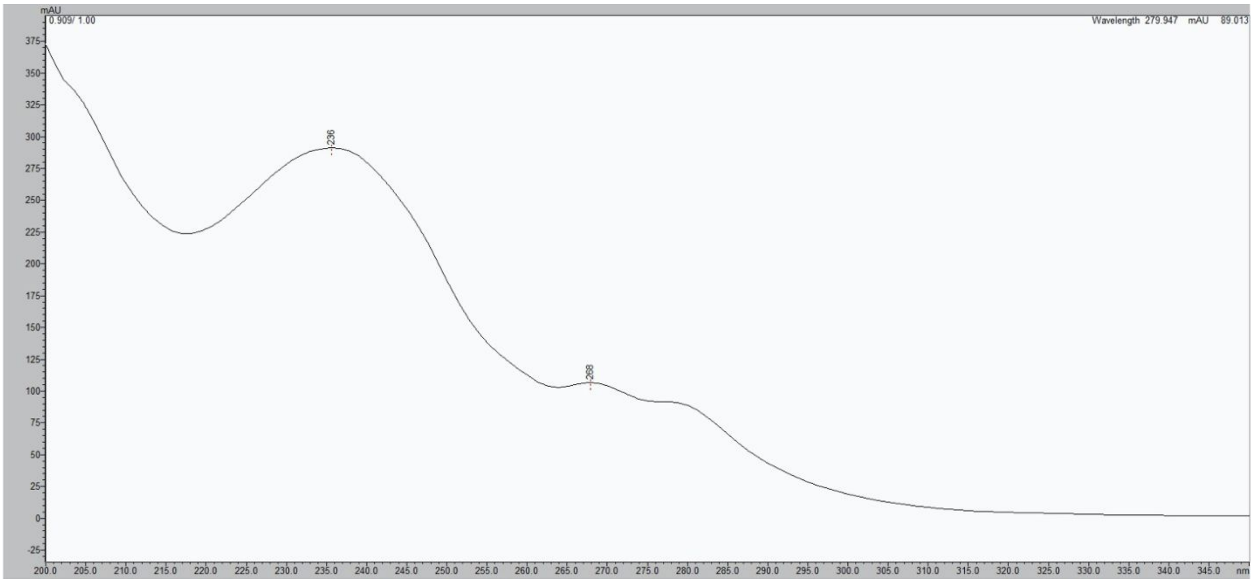

Figure S21: Crannenol B (**2**) UV λ_{max} (CH_3OH).

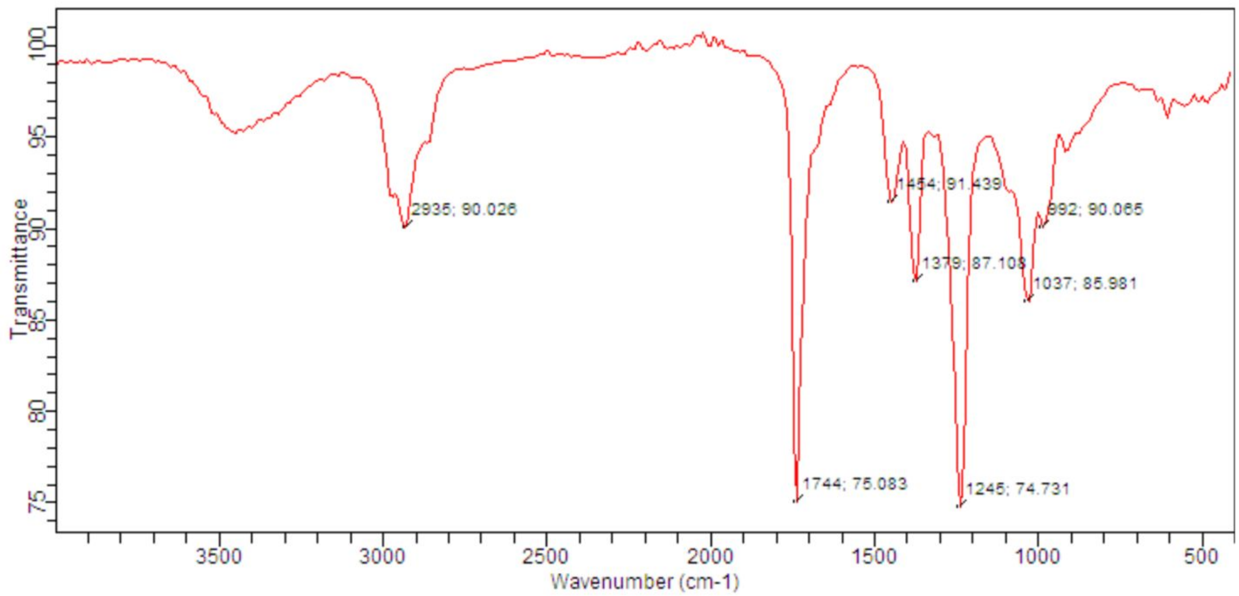

Figure S22: Crannenol B (**2**) IR spectrum (thin film).

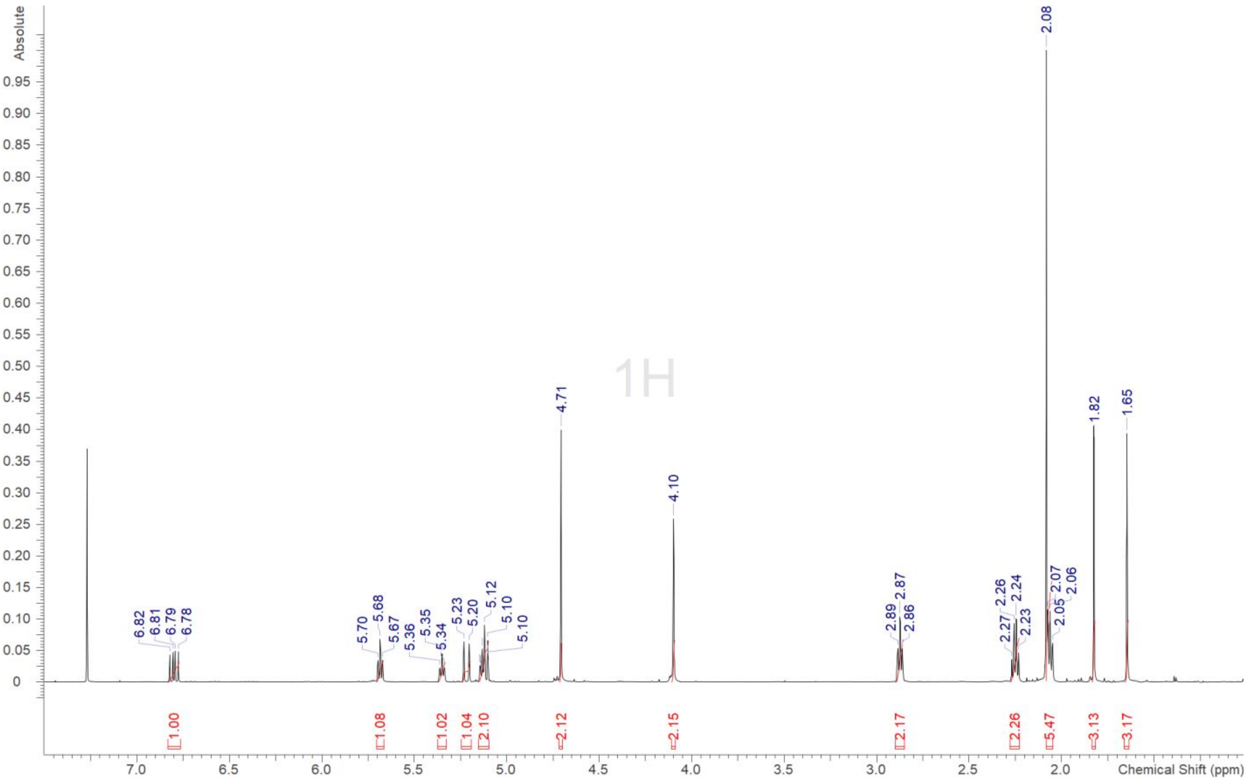

Figure S23: Crannenol C (**3**) ^{1}H NMR spectrum (600 MHz, $CDCl_3$).

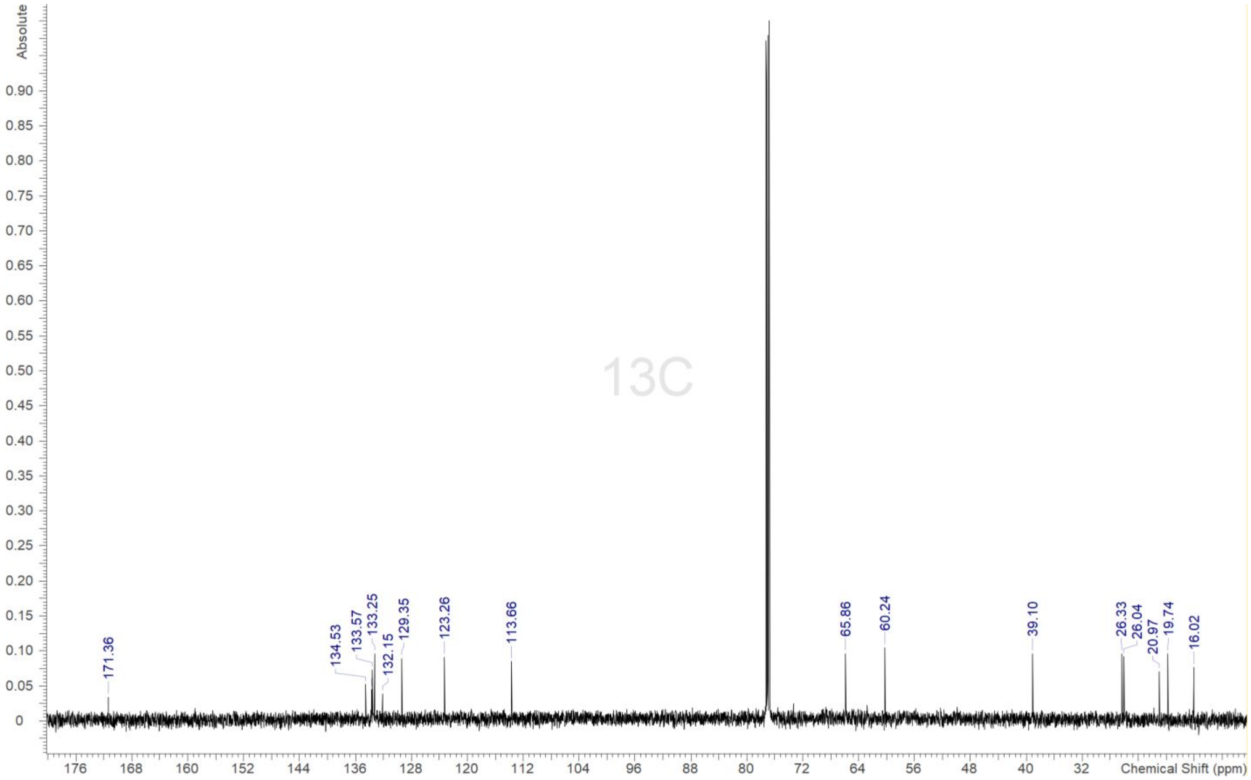

Figure S24: Crannenol C (**3**) ^{13}C NMR spectrum (150 MHz, $CDCl_3$).

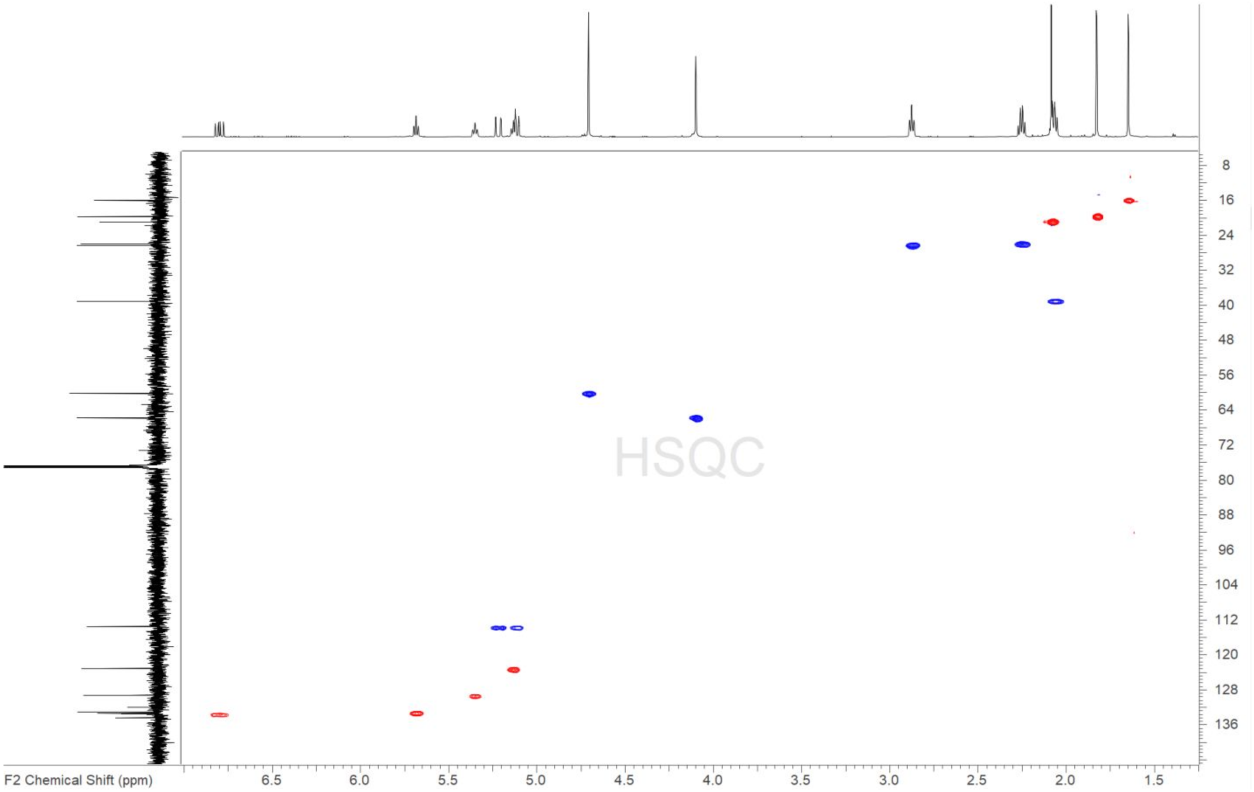

Figure S25: Crannenol C (**3**) HSQC NMR (500 MHz, $CDCl_3$).

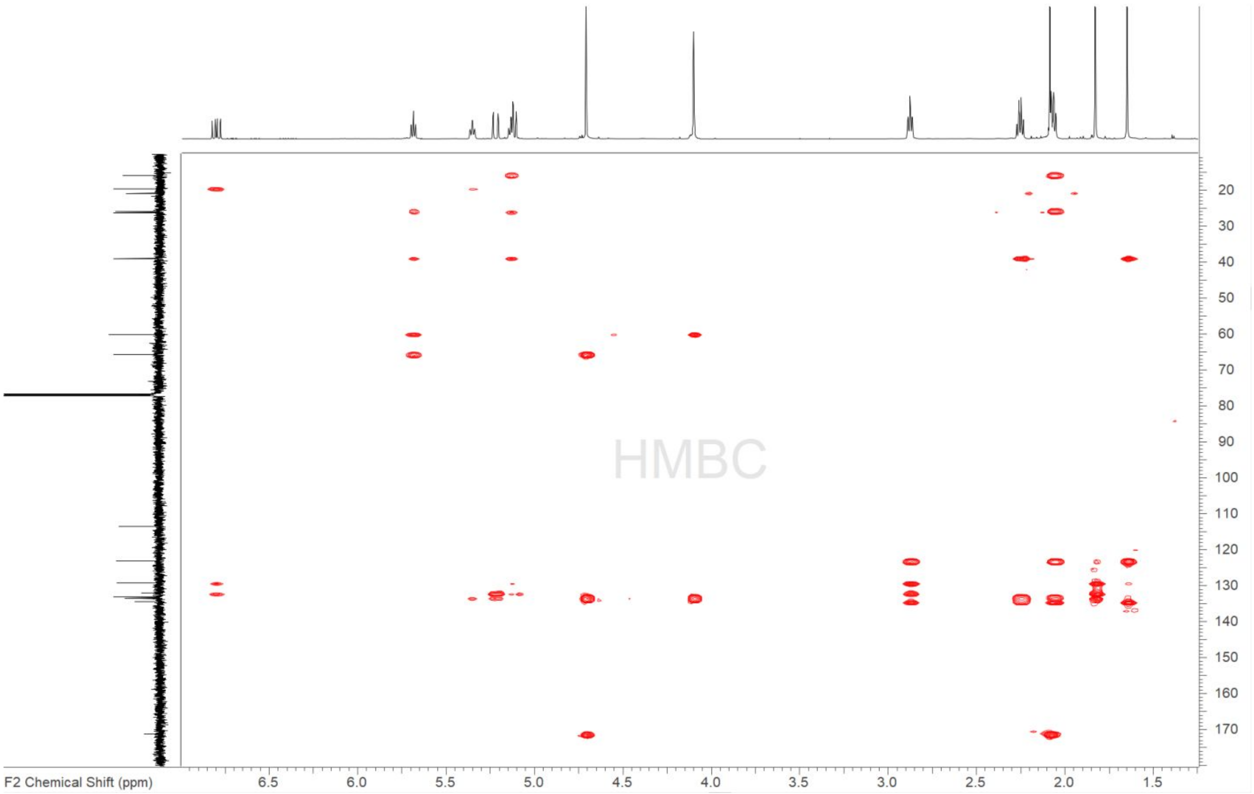

Figure S26: Crannenol C (**3**) HMBC NMR (500 MHz, $CDCl_3$).

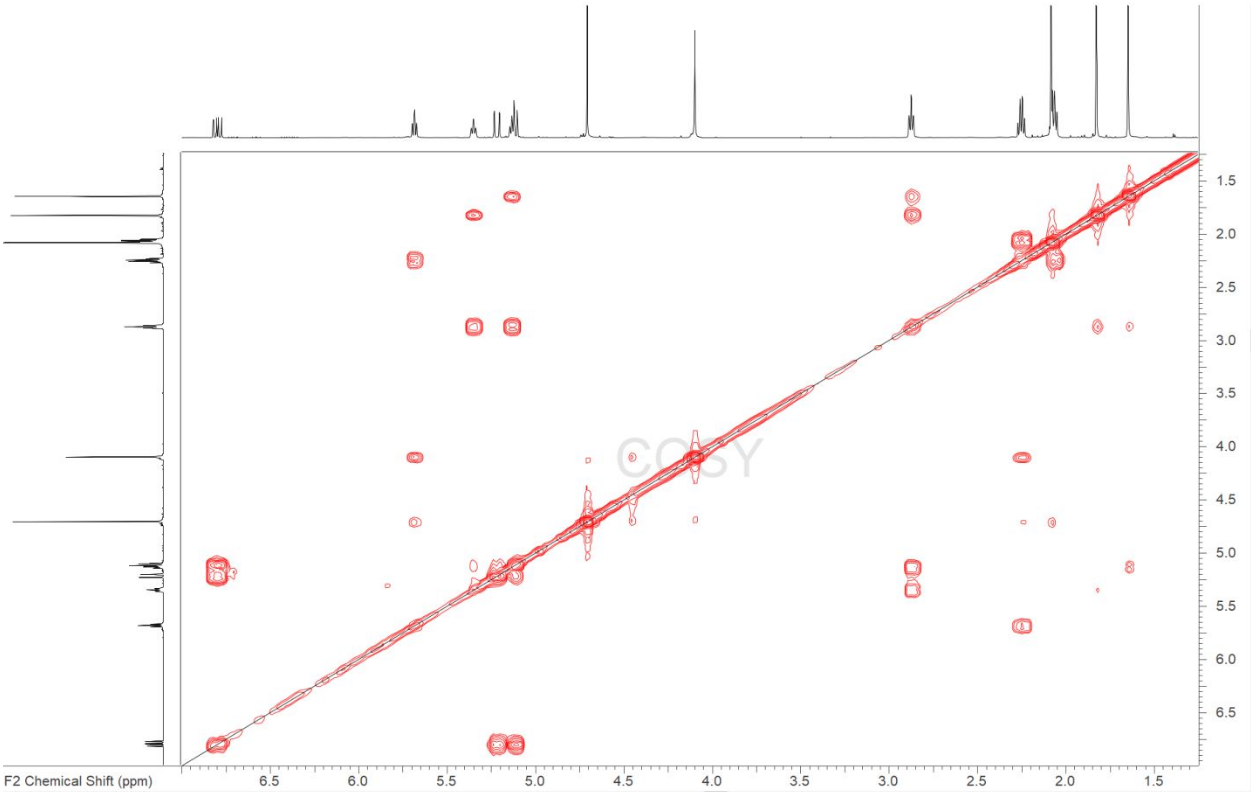

Figure S27: Crannenol C (**3**) COSY NMR spectrum (500 MHz, $CDCl_3$).

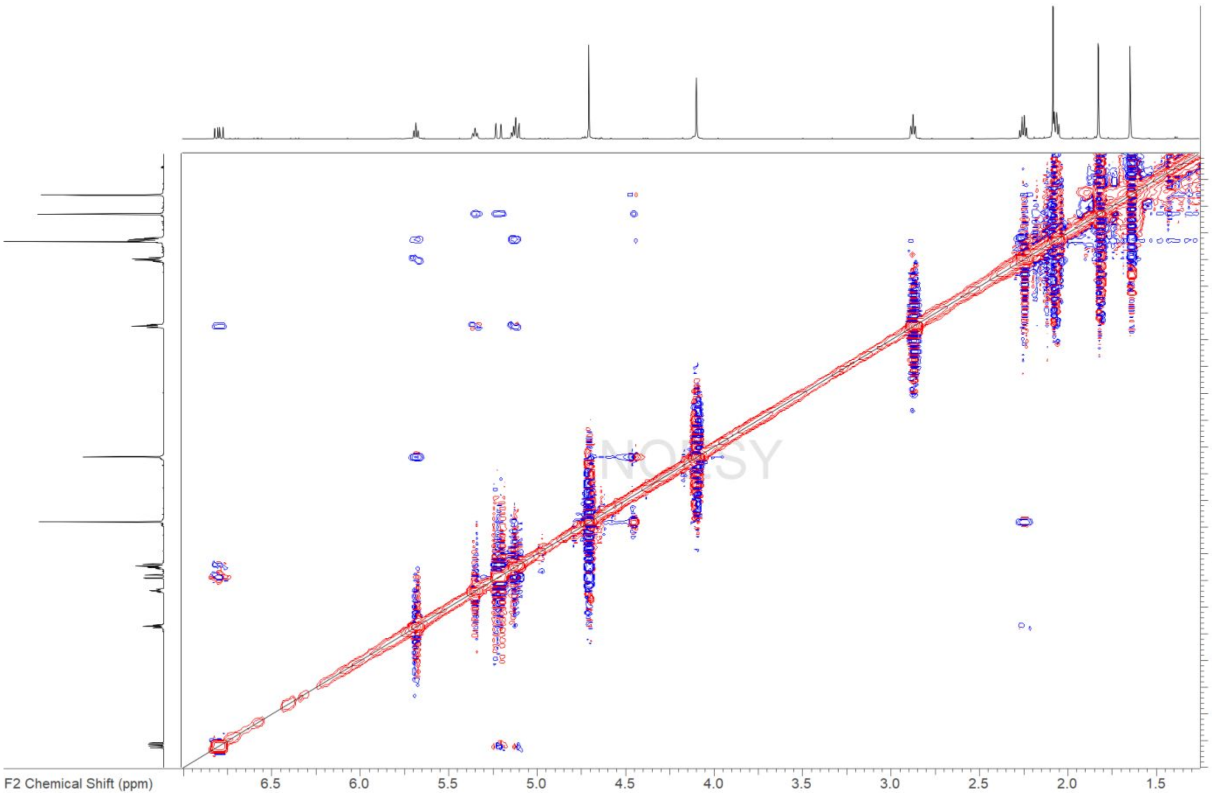

Figure S28: Crannenol C (**3**) NOESY NMR spectrum (500 MHz, $CDCl_3$).

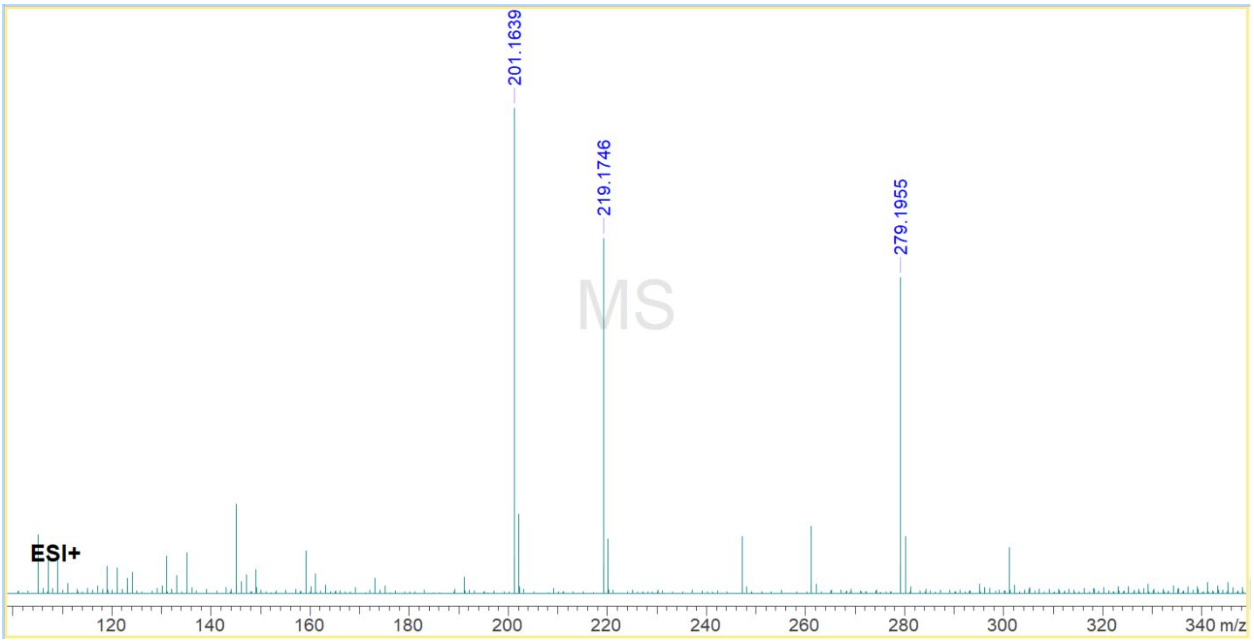

Figure S29: Crannenol C (**3**) HRESIMS.

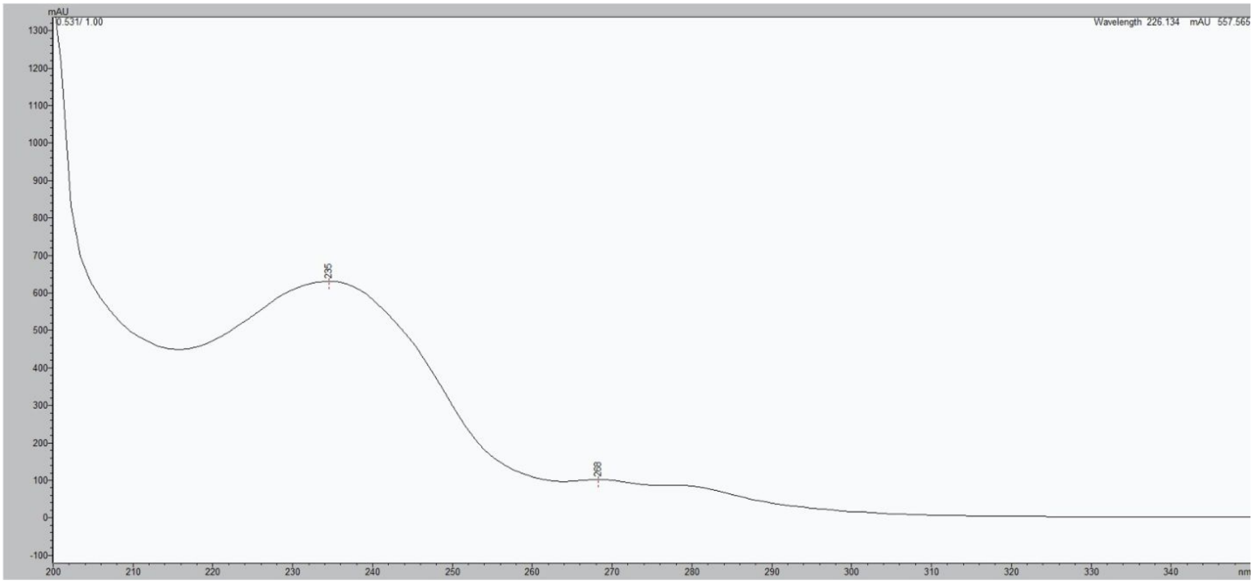

Figure S30: Crannenol C (**3**) UV λ_{max} (CH_3OH).

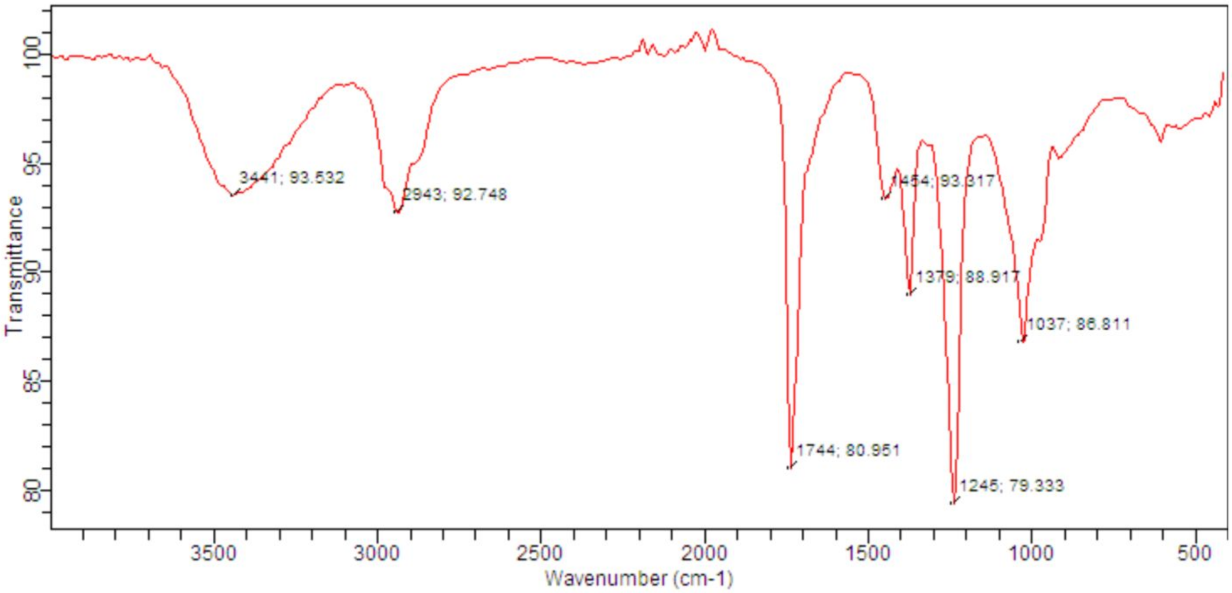

Figure S31: Crannenol C (**3**) IR spectrum (thin film).

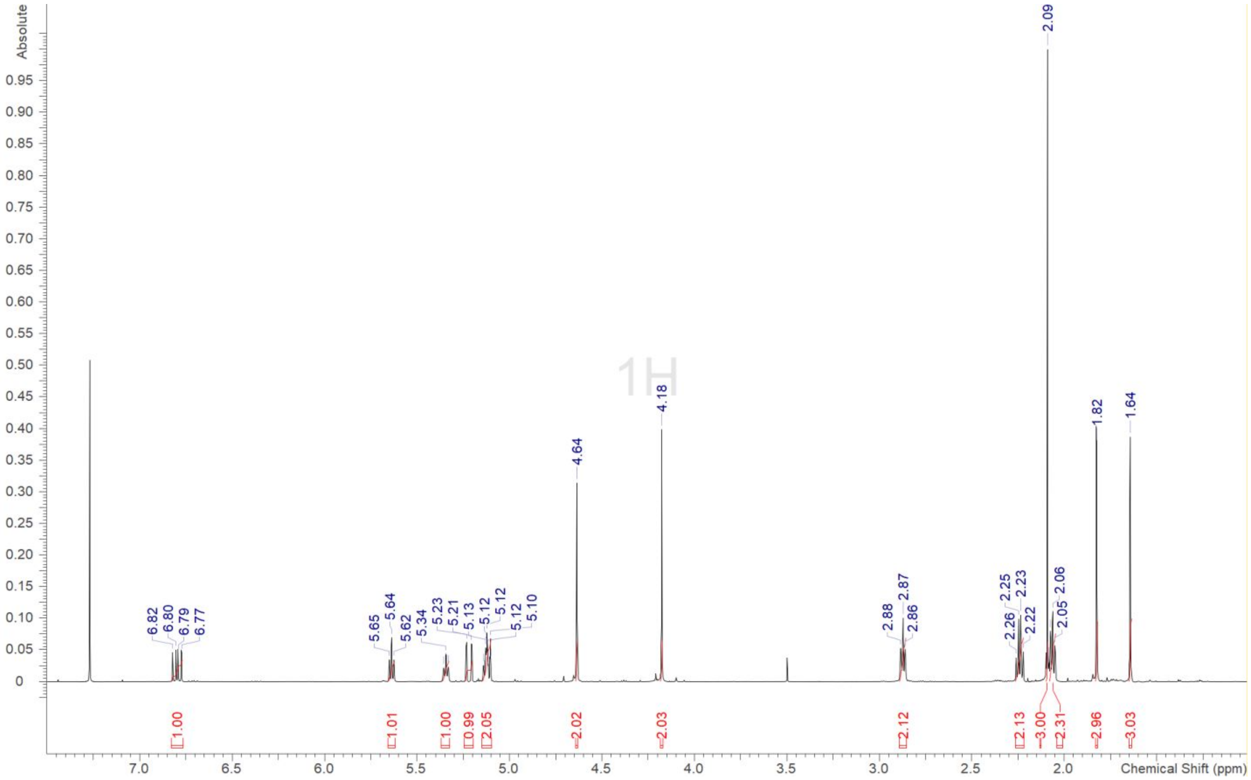

Figure S32: Crannenol D (**4**) ^{1}H NMR spectrum (600 MHz, $CDCl_3$).

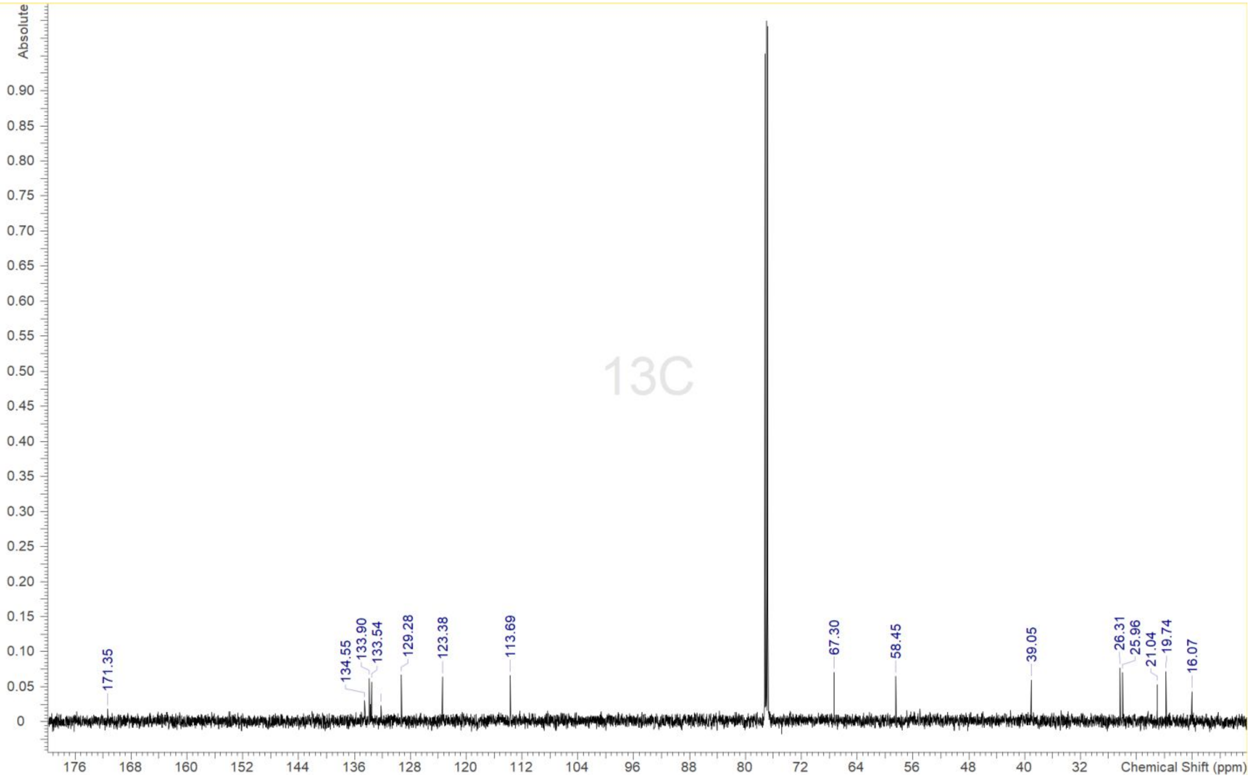

Figure S33: Crannenol D (**4**) ^{13}C NMR spectrum (150 MHz, $CDCl_3$).

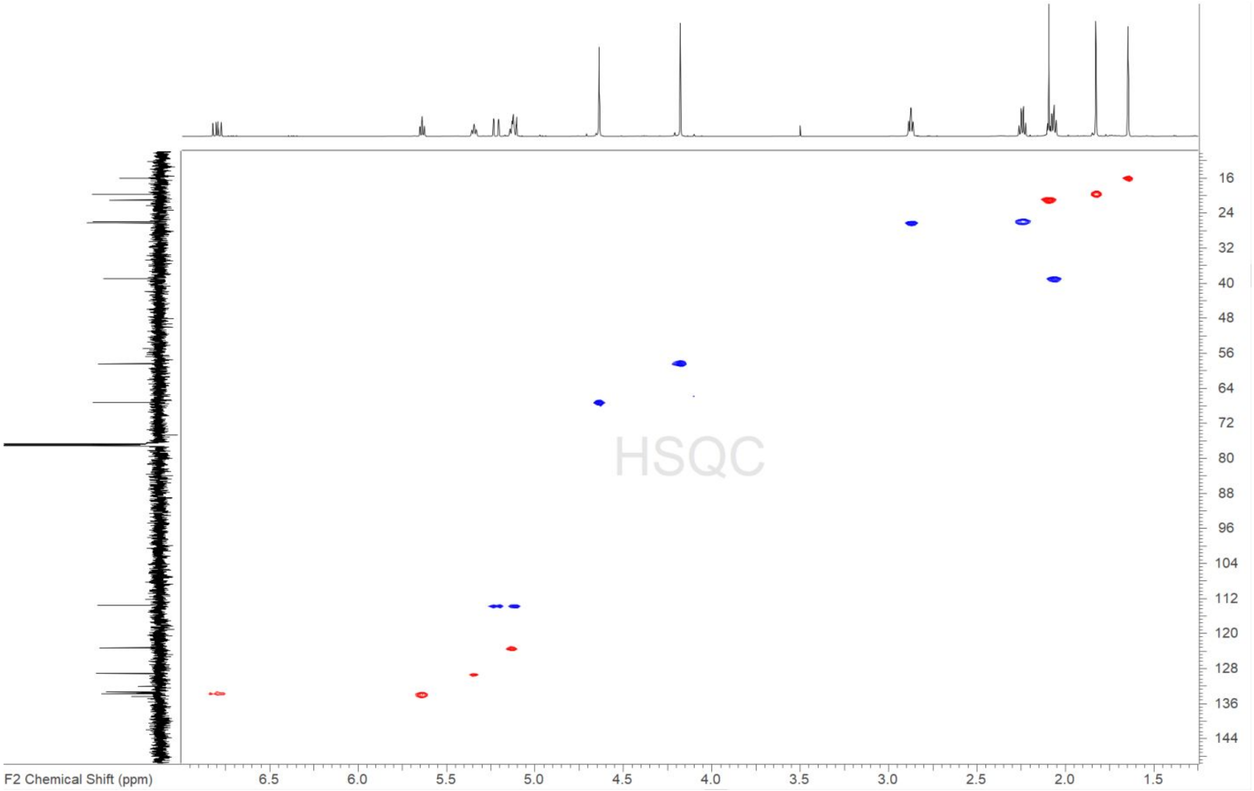

Figure S34: Crannenol D (**4**) HSQC NMR spectrum (500 MHz, $CDCl_3$).

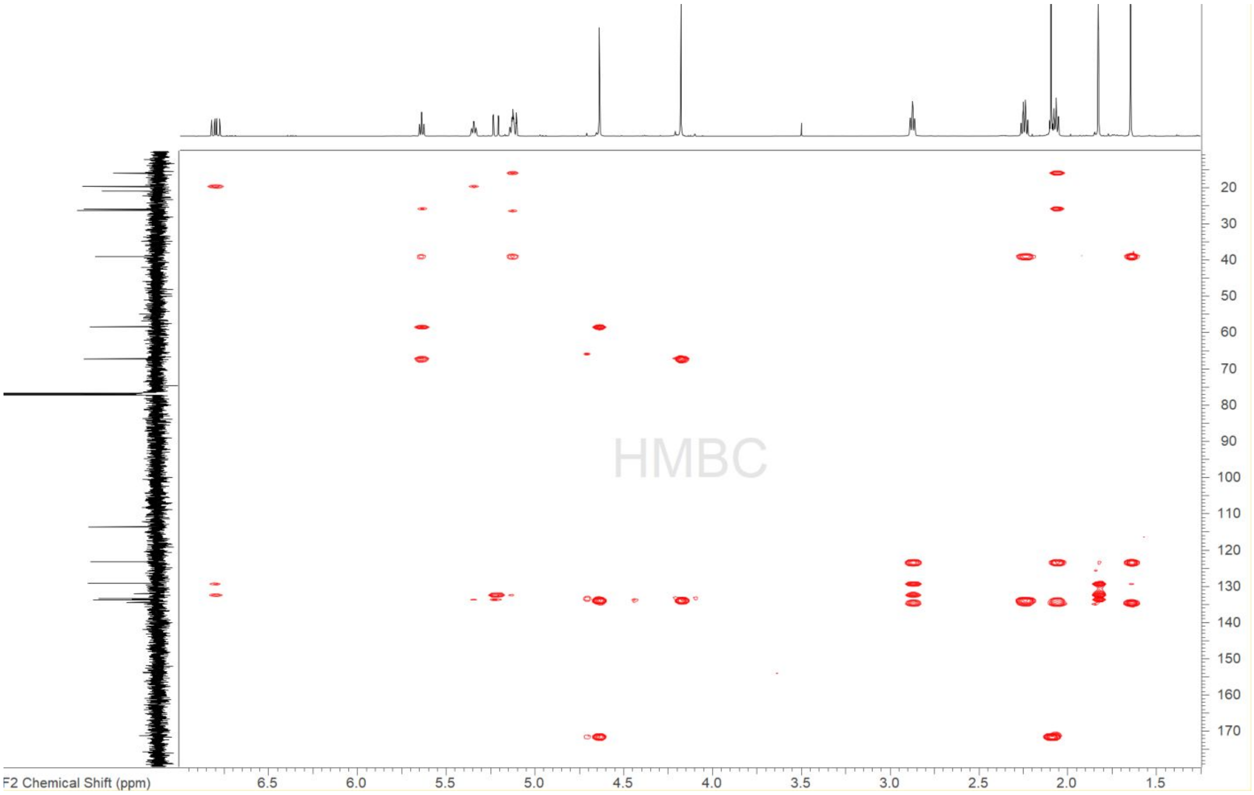

Figure S35: Crannenol D (**4**) HMBC NMR spectrum (500 MHz, $CDCl_3$).

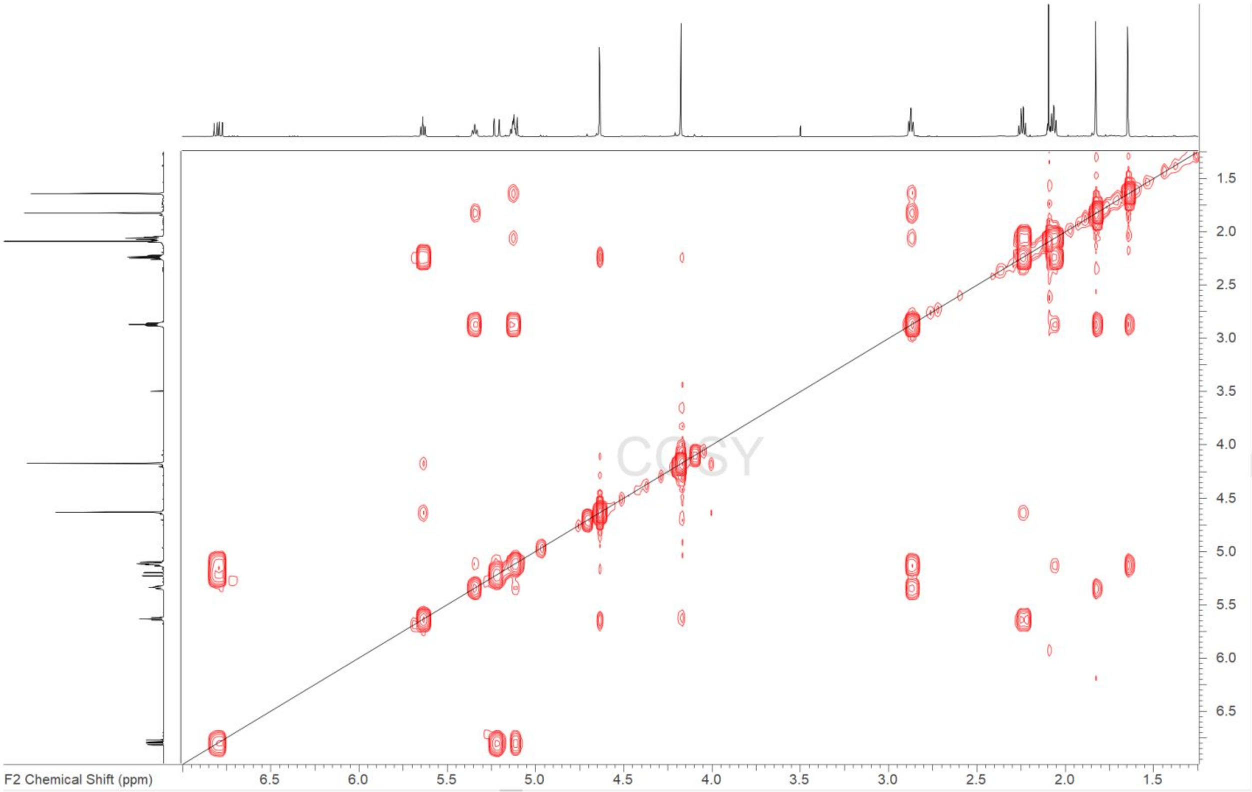

Figure S36: Crannenol D (**4**) COSY NMR spectrum (600 MHz, $CDCl_3$).

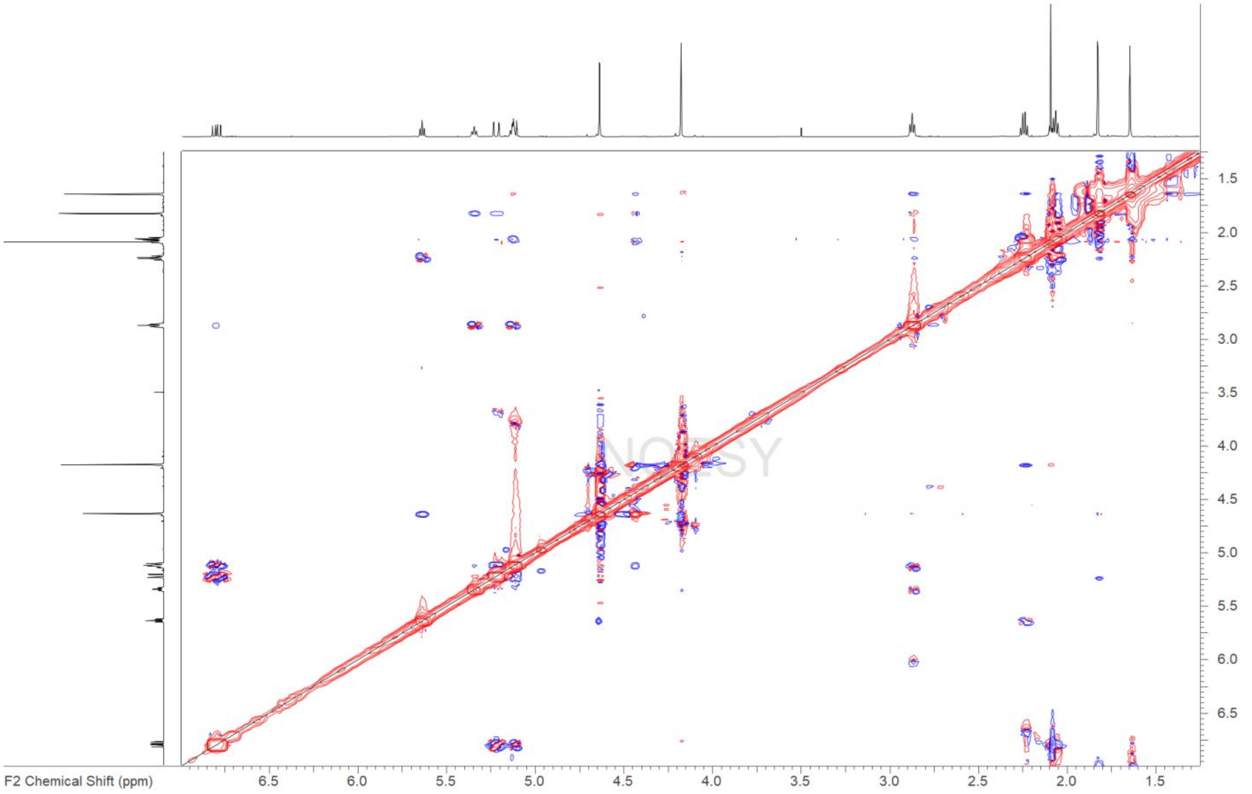

Figure S37: Crannenol D (**4**) NOESY NMR spectrum (500 MHz, $CDCl_3$).

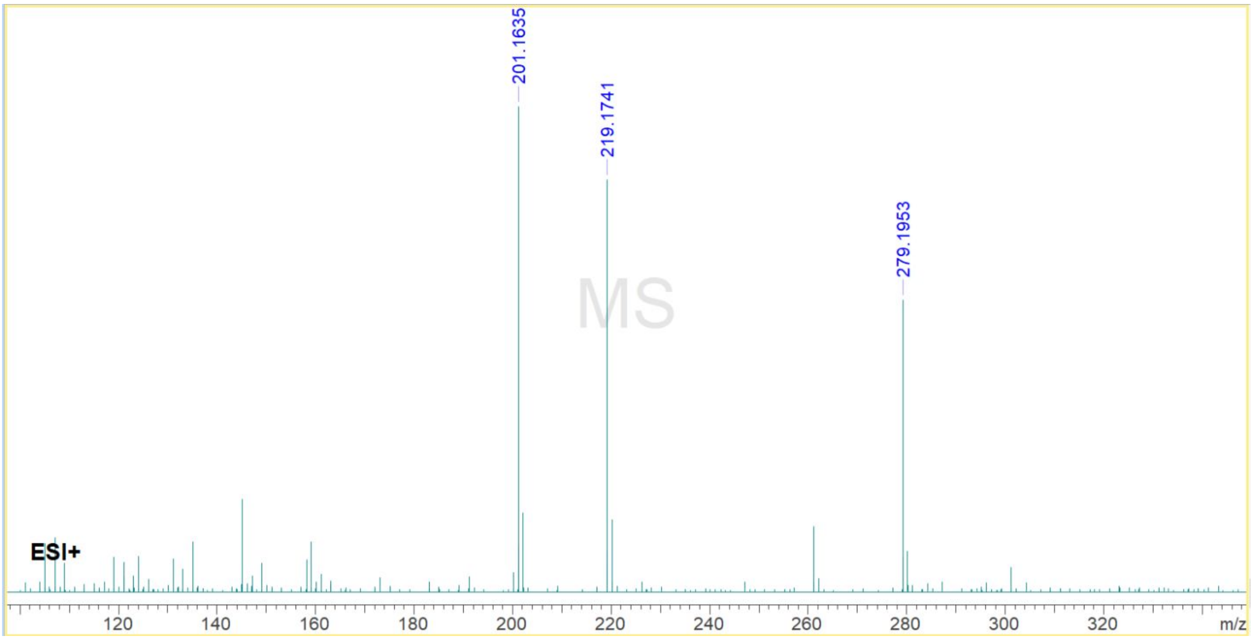

Figure S38: Crannenol D (**4**) HRESIMS.

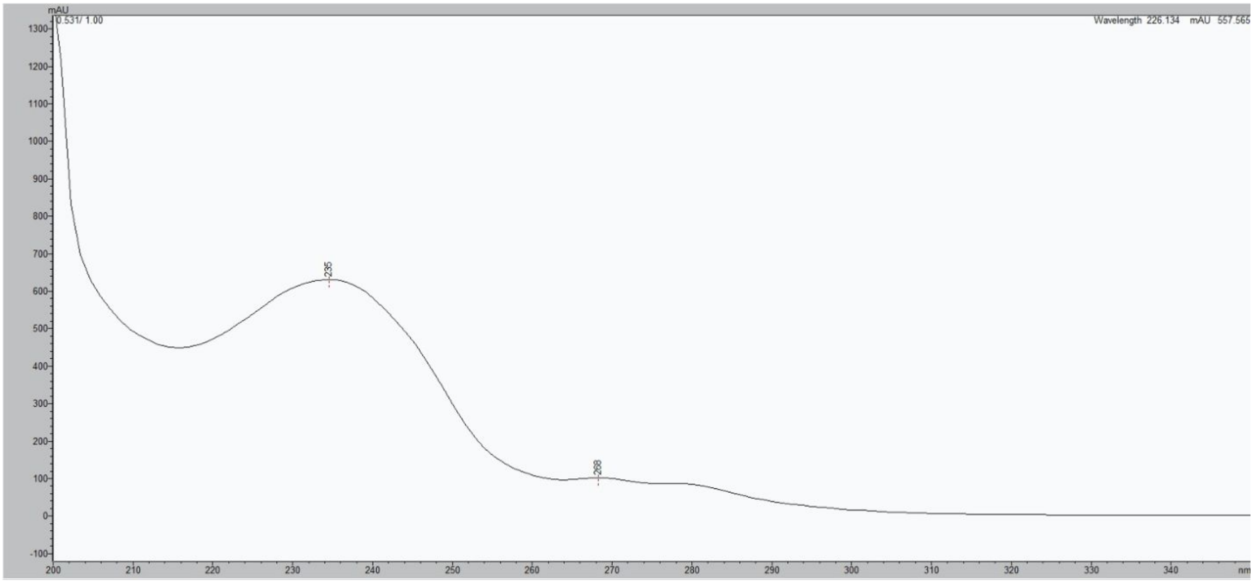

Figure S39: Crannenol D (**4**) UV λ_{max} (CH_3OH).

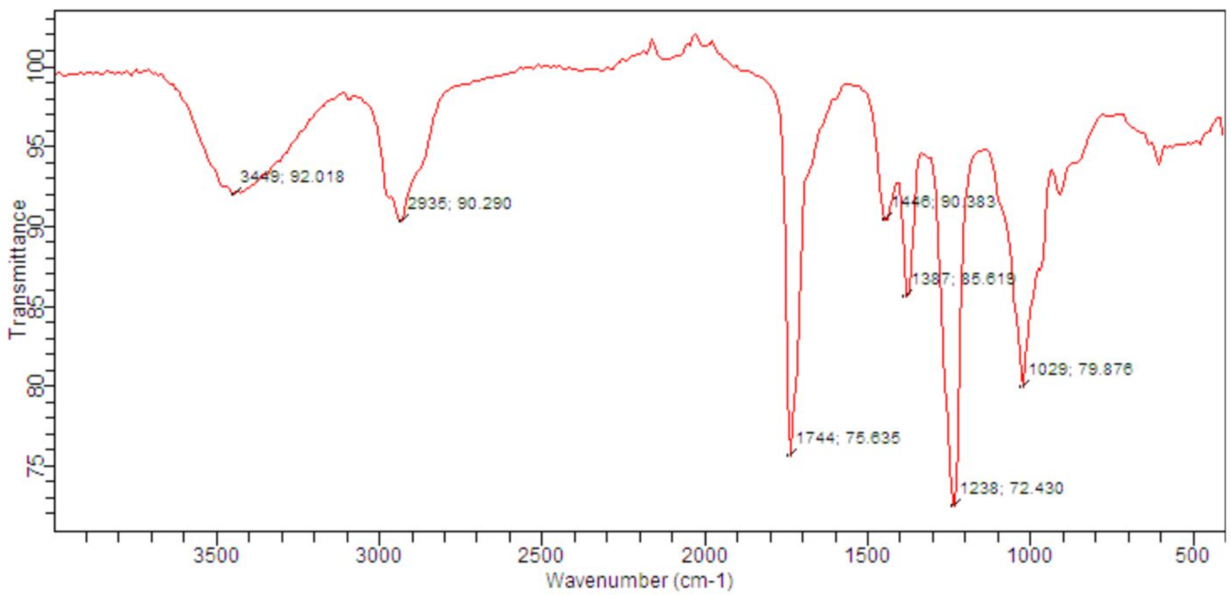

Figure S40: Crannenol D (**4**) IR spectrum (thin film).
